# Large‐Scale 2D Perovskite Nanocrystals Photodetector Array via Ultrasonic Spray Synthesis

**DOI:** 10.1002/adma.202417761

**Published:** 2025-02-19

**Authors:** Yoon Ho Lee, Won‐June Lee, Gang San Lee, Jee Yung Park, Biao Yuan, Yousang Won, Jungho Mun, Hanjun Yang, Sung‐Doo Baek, Haeun Lee, Joon Hak Oh, Timothy J. Pennycook, Gwangwoo Kim, Jianguo Mei, Letian Dou

**Affiliations:** ^1^ Davidson School of Chemical Engineering Purdue University West Lafayette IN 47907 USA; ^2^ James Tarpo Jr. and Magaret Tarpo Department of Chemistry Purdue University West Lafayette IN 47907 USA; ^3^ Department of Materials Science and Engineering Sungshin Women's University Seoul 01133 Republic of Korea; ^4^ Elmore Family School of Electrical and Computer Engineering Purdue University West Lafayette IN 47907 USA; ^5^ EMAT University of Antwerp Groenenborgerlaan 171 Antwerp 2020 Belgium; ^6^ School of Chemical and Biological Engineering Institute of Chemical Processes Seoul National University Seoul 08826 Republic of Korea; ^7^ POSCO‐POSTECH‐RIST Convergence Research Center for Flat Optics and Metaphotonics Pohang University of Science and Technology (POSTECH) Pohang 37673 Republic of Korea; ^8^ Department of Engineering Chemistry Chungbuk National University Cheongju 28644 Republic of Korea

**Keywords:** high‐performance photodetector array, large‐scale nanofabrication, perovskite nanocrystals, 2D materials, ultrasonic spray synthesis

## Abstract

2D perovskite (PVSK) single crystals have received significant attention due to their unique optical and optoelectronic properties. However, current synthesis methods face limitations, particularly in large‐area fabrication, which remain critical barriers to practical applications. In this study, the synthesis of red/green/purple‐blue‐colored 2D PVSK nanocrystals over a large area (4‐inch wafer) and the fabrication of high‐performance photodetector arrays are presented via a facile yet efficient spray‐coating approach with a liquid‐bridge transport effect. The photodetector array achieves 100% working yield, high photo‐responsivity (1.5 × 10^6^ A W^−1^) and specific‐detectivity (1.1 × 10^16^ Jones) with competitive photomapping characteristics. An intelligent vision system for automatic shape recognition is further demonstrated with a recognition rate exceeding 90%. This study provides significant advances in the scalable synthesis of nanoscale 2D PVSK crystals, their integration into large‐area optoelectronic devices, and their potential use in artificial‐intelligence systems.

## Graphical abstract

Red, green, and blue (R/G/B) colored 2D perovskite nanocrystals synthesized via an ultrasonic spray method demonstrate outstanding photo‐detection performance in 4‐inch photodetector arrays with a 100% operational yield, enabled by a liquid‐bridge‐transport strategy. This scalable, self‐aligned synthesis provides a practical pathway for high‐performance, large‐area perovskite optoelectronic devices and shows promising potential for broader applications in integrated optoelectronic systems.

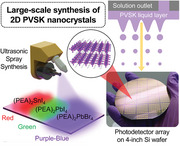

## 1 Introduction

Research on electronics using 2D materials, such as graphene, hexagonal boron nitride (*h*‐BN), transition metal dichalcogenides (TMDs), transition metal oxides (TMOs), and MXenes, has advanced significantly in recent years, driven by their unique electronic and mechanical properties with defect‐free interfaces, leading to extensive exploration of their conductor, semiconductor, and insulator characteristics and their potential applications in electronic devices.^[^ ^1^, ^2^, ^3^, ^4^ ^]^ For practical industrial commercialization, there has been a notable increase in studies on fabricating large‐area electronics arrays using coating methods such as slot‐die, blade, and screen printing with 2D material inks that are pre‐synthesized and dispersed in solution.^[^ ^1^, ^5^, ^6^, ^7^ ^]^

Among 2D materials, 2D organic‐inorganic hybrid perovskite (PVSK) single crystals represent a new class of semiconductors that exhibit exceptional optoelectronic properties, tunable chemistry, and notable environmental stability, making them highly attractive for various applications.^[^ ^8^, ^9^, ^10^, ^11^, ^12^, ^13^ ^]^ Studies have demonstrated the high‐performance potential of 2D PVSK single crystals in applications such as solar cells, photodetectors (PDs), transistors, and memory devices. However, research on array devices remains limited, largely due to the considerable challenges in achieving high‐density integration and scaling up.^[^ ^10^, ^14^, ^15^, ^16^ ^]^

The first hurdle is the absence of a synthetic method for synthesizing nanometer‐sized 2D PVSK single crystals over a large area. Conventional synthesis methods for thin 2D PVSK single crystals, including solution drying, mechanical exfoliation, epitaxial growth, and interface‐confinement growth, reported the synthesis of only micrometer‐sized 2D PVSK single crystals due to the challenges in forming small‐sized solution droplets or synthesizing bulk crystals of PVSK at a small scale. Furthermore, covering the substrate for the synthesis of crystals distributed over a large area requires a substantial amount of solution or a significant number of exfoliation and transfer processes, posing a significant drawback to these methods.

The second hurdle lies in the methodological absence of fabricating high‐performance devices that enable the positioning of 2D PVSK single nanocrystals between electrodes and implementing them as an array over a large area with minimal crystal damage during the connection process between the electrodes and crystals.^[^ ^17^, ^18^, ^19^ ^]^ Conventional synthesis methods are limited in that 2D PVSK single microcrystals synthesized through these approaches tend to be positioned or transferred randomly to various locations.^[^ ^10^, ^20^, ^21^, ^22^, ^23^, ^24^ ^]^ This randomness reduces the likelihood of crystals being positioned between electrodes at a specific location across a wide area, ultimately resulting in a lower device working yield.

Here, we report a facile yet effective spray‐coating method for synthesizing 2D PVSK nanocrystals and demonstrate its application in the high‐yield production of wafer‐scale PD arrays. 2D PVSK single nanocrystals were successfully synthesized using ultrasonic‐assisted spray coating (USSC) technology, allowing uniform coating of micron‐sized PVSK solution droplets onto the substrate. It has also been confirmed that the size of the crystals can be adjusted to tens to hundreds of nanometers by controlling the coating conditions. 2D PVSK nanocrystals were also successfully synthesized through a USSC on various substrates, including Si wafer, glass, indium tin oxide (ITO), metal foil, and Teflon film, highlighting the broad applicability of this approach. Moreover, this methodology proved to be versatile across an extensive range of 2D halide PVSK species, showing various compositions for red, green, and purple‐blue color emissions.

Moreover, ultraviolet (UV)‐PDs‐based 2D PVSK single nanocrystals exhibit a remarkably high photoresponsivity (*R*) of 1.5 × 10^6^ A W^−1^ and a specific detectivity (*D* ^*^) of 1.1 × 10^16^Jones. To the best of our knowledge, our 2D PVSK single nanocrystal PDs showed the highest *D* ^*^ for the highest *R* in comparison with other 2D and 3D PVSK PDs reported so far. Furthermore, a 4‐inch wafer‐scale and 19 × 19 PD array were fabricated in this manner, demonstrating an impressive 100% yield with high‐resolution photomapping characteristics. This was achieved by employing liquid‐bridge transport and droplet‐pinned crystallization effects, promoting predominant crystal synthesis between the electrodes. Additionally, we demonstrated a machine learning‐based intelligent vision system for smart automatic shape recognition, achieving a high recognition rate exceeding 90%. The direct synthesis of large‐area distributed 2D PVSK nanocrystals, along with the associated device fabrication methods, provides a novel platform for probing the fundamental electronic and optical properties of PVSK materials. This integration of the material synthesis and optoelectronics development represents a significant advancement toward the realization of integrated electronic systems based on 2D materials.

## 2 Results and Discussion

### 2.1 Synthesis of 2D PVSK Nanocrystals Using USSC

**Figure 1:**
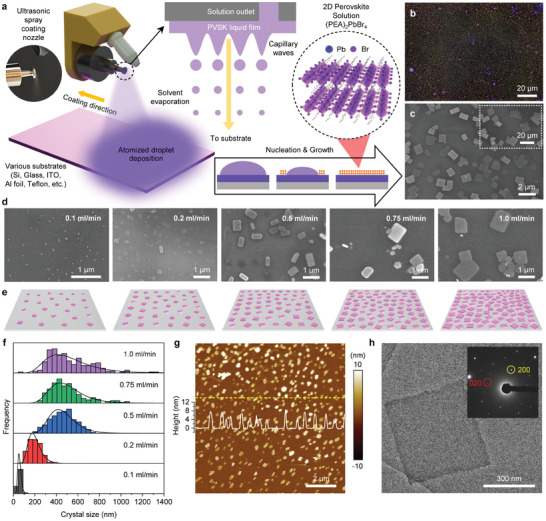
USSC for synthesis of (PEA)_2_PbBr_4_ 2D PVSK single nanocrystals. a) Schematic illustration of an automated USSC for the synthesis of (PEA)_2_PbBr_4_ 2D PVSK single nanocrystals (left inset: ultrasonic spray nozzle during the atomized liquid dispensing). b) PL‐OM and c) SEM image of (PEA)_2_PbBr_4_ nanocrystals on Si/SiO_2_ wafers based on 0.5 mL min^−1^ spray nozzle flow rates. d) SEM images of (PEA)_2_PbBr_4_ nanocrystals on Si/SiO_2_ wafers according to the spray nozzle flow rates from 0.1 to 1.0 mL min^−1^. e) Schematic illustration of nanocrystal size variation at different flow rates. f) Statistical size distributions of the (PEA)_2_PbBr_4_ nanocrystals at different flow rates. g) AFM surface morphology image (inset: cross‐sectional profile for the synthesized 2D PVSK crystals) based on 0.5 mL min^−1^ flow rates h) HR‐TEM image of 2D (PEA)_2_PbBr_4_ nanocrystals on TEM grids (inset: SAED pattern image of 2D single PVSK crystal).

Schematic images of the USSC system and the growth of 2D PVSK single nanocrystals are shown in **Figure** **1**a (see details in the Experimental Section). First, an optimized phenethylammonium (PEA)‐based (PEA)_2_PbBr_4_ cocktail solution was loaded into the spray coating equipment and fed into the nozzle at a constant dispensing rate. During this process, the solution was initially atomized into micron‐sized droplets using an ultrasonic nozzle. Subsequently, it was propelled downward by inert nitrogen (N_2_) gas flow (Figure S1, Supporting Information), resulting in their widespread dispersion of these droplets onto the heated substrate, where they rapidly evaporated. Concurrently, as the solvent evaporates, the nucleation and growth of 2D PVSK crystals take place on the substrate. First, we synthesized (PEA)_2_PbBr_4_ nanocrystals on a Si/SiO_2_ wafer substrate. The photoluminescence (PL)‐optical microscopy (OM) image and scanning electron microscopy (SEM) image of (PEA)_2_PbBr_4_ nanocrystals based on 0.5 mL min^−1^ solution dispensing rate are shown in Figure 1b,c, respectively. The corresponding OM and low‐magnification SEM images are also shown in Figures S2 and S3 (Supporting Information). The rectangular‐shaped (PEA)_2_PbBr_4_ nanocrystals grew very uniformly and densely on the Si wafer substrate, and the PL‐OM image also effectively shows the purple‐blue‐colored PL characteristics of the (PEA)_2_PbBr_4_ crystals.

The crystal size can also be effectively controlled by adjusting dispensing rates in the USSC system. Figure 1d,e shows the SEM and schematic images of (PEA)_2_PbBr_4_ crystals as a function of the dispensing rate. The OM and PL‐OM images as a function of the dispensing rate are also shown in Figure S4 (Supporting Information). The higher the dispensing rate, the greater the number of micron‐sized droplets being coated, and consequently, larger‐sized droplets are formed on the substrate by coarsening of small‐sized droplets, leading to an increase in crystal size.^[^ ^25^, ^26^ ^]^ The size distributions and number density of the crystals were determined by scanning electron microscopy (SEM) analysis, as shown in Figures 1f and S5 (Supporting Information). By adjusting the dispensing rates (0.1, 0.2, 0.5, 0.75, and 1.0 mL min^−1^), the size of the (PEA)_2_PbBr_4_ crystal can be tuned within the range of ≈50 nm to 1 µm. As the dispensing rate increases, the crystal size initially grows linearly up to a point (≈0.5 mL min^−1^). However, beyond this rate, the increase in crystal size gradually slowed, indicating that the droplet size on the substrate remained relatively stable after reaching the dispensing rate threshold. Exceeding the critical dispensing rate for 2D PVSK crystal formation (above 1.0 mL min^−1^) would cause the solution to fully saturate the entire substrate (i.e., wet mode) and lead to the synthesis of irregular micro‐sized and/or coarsened rather than nanosized crystals.^[^ ^25^ ^]^ As the dispensing rate increased from 0.1 to 1.0 mL min^−1^, the number of crystals decreased from 1 quadrillion to 200 billion per cm^2^, with the rate of decrease gradually slowing in a similar pattern to the rate of crystal size increase. It is noted that average sizes of the 2D perovskite crystals synthesized by conventional solvent drying method are 3.9 µm, which are several to dozens of times larger and exhibits lower uniformity compared to the crystals synthesized via the USSC process (Table S1 and Figure S6, Supporting Information).

In the atomic force microscopy (AFM) images, the height profile of the (PEA)_2_PbBr_4_ nanocrystals under the 0.5 mL min^−1^ dispensing condition appeared to be very thin, with a thickness ranging from 5 to 7 nm, indicating that they are either bilayers or tri‐layers (Figure 1g). The thickness of the synthesized crystals increased linearly from 3.1 to 20.9 nm as the dispensing rate increased from 0.1 to 1 mL min^−1^ (Figures S7 and S8, Supporting Information).

The high‐resolution transmission electron microscopy (HR‐TEM) image reveals a uniform surface and flawless rectangular morphology of the (PEA)_2_PbBr_4_ nanocrystal (Figure 1h). In the selected area electron diffraction (SAED) pattern, similar to the XRD pattern, sharper peaks signify a better crystallinity of the crystal (Figures 1h and S9, Supporting Information). The electron diffraction patterns obtained from the experiment show sharp patterns, indicating that the synthesized crystal possesses excellent crystallinity. The crystal planes corresponding to the diffraction were identified based on the simulated diffraction pattern (Figure S10, Supporting Information). The dynamical electron diffraction was simulated with py4DSTEM, which is consistent with the experimental diffraction patterns.

To demonstrate the broad applicability of crystal synthesis through USSC, we synthesized 2D PVSK crystals not only on Si wafer substrates but also on various types of substrates, including glass, ITO, aluminum foil, and Teflon film substrates. Figure S11, Supporting Information, shows a series of photographs, OM, PL‐OM, and SEM images of (PEA)_2_PbBr_4_ crystals synthesized via USSC on glass, ITO, aluminum foil, and Teflon film substrates at 0.5 mL min^−1^ dispensing rate. All crystals were synthesized in a square pattern on a large substrate exceeding 50 cm^2^, exhibiting sharp PL characteristics with a distinct purple‐blue emission, indicating the successful synthesis of (PEA)_2_PbBr_4_ single nanocrystals. The distributions of the crystal sizes on these substrates are shown in Figure S12 (Supporting Information). The variation in the average crystal size depending on the substrate type indicates that, due to the characteristics of the USSC based on the solution process, the coating properties of micron‐sized droplets on the substrate surface differ depending on the surface energy and surface morphology.

It is noted that ≈700 billion crystals were synthesized per 1 cm^2^ on SiO_2_, glass, ITO, and Al foil substrates, demonstrating the ability to uniformly mass‐produce a vast number of nanocrystals across various substrates. On the Teflon substrate, ≈200 billion crystals were synthesized per square centimeter, a slightly lower count compared to the other substrates, yet still represents a substantial number. This reduction may be due to the poor adhesion between the PVSK precursor solution and the Teflon surface during USSC, attributable to the low surface energy of the Teflon substrate. These number of crystals per 1 cm^2^ in various dispensing rate and types of substrate are summarized in Table S2 (Supporting Information).

The PL (Figure S13a, Supporting Information) and X‐ray diffraction (XRD) spectra (Figure S13b, Supporting Information) for the (PEA)_2_PbBr_4_ crystals on various substrate types exhibited distinct characteristic peaks of (PEA)_2_PbBr_4_, confirming the high crystallinity and quality of the crystals.^[^ ^21^ ^]^

**Figure 2:**
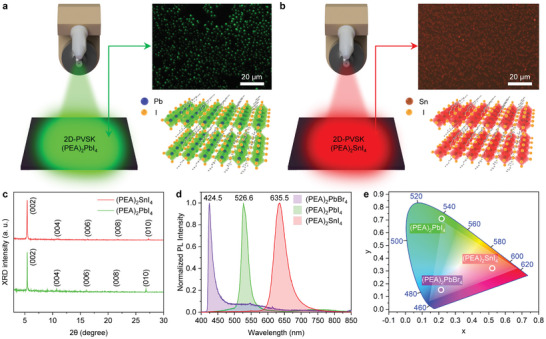
USSC for synthesis of (PEA)_2_PbI_4_ and (PEA)_2_SnI_4_ 2D PVSK single nanocrystals. Schematic illustration of the USSC and crystal structures with PL‐OM images for a) (PEA)_2_PbI_4_ and b) (PEA)_2_SnI_4_ nanocrystals synthesized by USSCs. c) XRD results for (PEA)_2_PbI_4_ and (PEA)_2_SnI_4_ nanocrystals synthesized by USSCs. d) PL spectrum and e) CIE (CIE 1931) color coordinates corresponding to the blue‐purple (PEA)_2_PbBr_4_, green (PEA)_2_PbI_4,_ and red (PEA)_2_SnI_4_ single nanocrystals (NTSC standard: bright area in Figure 2e).

To explore the feasibility of USSC‐based 2D PVSK nanocrystal synthesis for different PL colors, we synthesized (PEA)_2_PbI_4_ and (PEA)_2_SnI_4_, which exhibited green and red‐colored PL characteristics, respectively. **Figure** **2**a,b shows schematic illustrations of the USSC and crystal structures with their PL‐OM images of (PEA)_2_PbI_4_ and (PEA)_2_SnI_4_, respectively. Both (PEA)_2_PbI_4_ and (PEA)_2_SnI_4_ crystals show clear, uniformly distributed square shapes with distinct green‐ and red‐colored PL characteristics, respectively. The XRD patterns of these 2D crystals shown in Figure 2c align well with those reported in previous studies.^[^ ^27^, ^28^ ^]^ Figure 2d,e shows the PL spectra of (PEA)_2_PbBr_4_, (PEA)_2_PbI_4_, and (PEA)_2_SnI_4_ 2D PVSK nanocrystals and the International Commission on Illumination (CIE) chromaticity diagram with the color coordinates of the nanocrystals highlighted, respectively. By varying the types of inorganic and halide elements in 2D PVSK materials using the USSC approach, crystals exhibiting three primary colors of light are synthesized: purple‐blue (424 nm) for (PEA)_2_PbBr_4_, green (526 nm) for (PEA)_2_PbI_4_, and red (635 nm) for (PEA)_2_SnI_4_. Figure S14 (Supporting Information) shows the UV–vis absorbance spectra for the (PEA)_2_PbBr_4_, (PEA)_2_PbI_4_, and (PEA)_2_SnI_4_ 2D PVSK nanocrystals, clearly demonstrating significant differences in absorption depending on the type of crystal. It is also noted that the crystals synthesized using our USSC method are significantly smaller and exhibit a more uniform distribution on the substrate compared to those synthesized using the conventional solvent drying method, which involves dropping the same precursor solution and annealing the crystals (Figures S6,S15 and S16, Supporting Information). In addition, spin‐coating the perovskite precursor solutions results in the formation of 2D perovskite clusters on the film, which are not uniform or dense, rather than square‐shaped crystals (Figure S17, Supporting Information).

### 2.2 Liquid Bridge Transport and Droplet‐Pinned Crystallization

To further investigate the advantages of the USSC, we fabricated (PEA)_2_PbBr_4_ UV photodetectors by synthesizing 2D PVSK crystals using a pre‐prepared bottom electrode via the USSC method. The USSC method, with its utilization of micro‐sized droplets to leverage liquid confinement and transport effects within microstructures, allows for more nanocrystal positioning between electrodes, resulting in exceptionally high device production yields, whereas the conventional method of nanocrystal synthesis through solution dropping and evaporation, characterized by random crystal placement, has a limitation of nanocrystal positioning, leading to lower device production yields.

The electrodes with a small gap of 200 nm and contact pads (200 µm gap) connected to the electrodes were prepared by electron beam (e‐beam) lithography and photolithography, respectively, followed by thermal deposition of Ti/Au. The details of the fabrication process are described in the Experimental Section and Figure S18 (Supporting Information). The OM images of only electrodes and electrodes with contact pads and the SEM image of electrodes, indicating successful fabrication of both the electrode and the contact pads, are shown in Figures S19 and S20 (Supporting Information), respectively.

**Figure 3:**
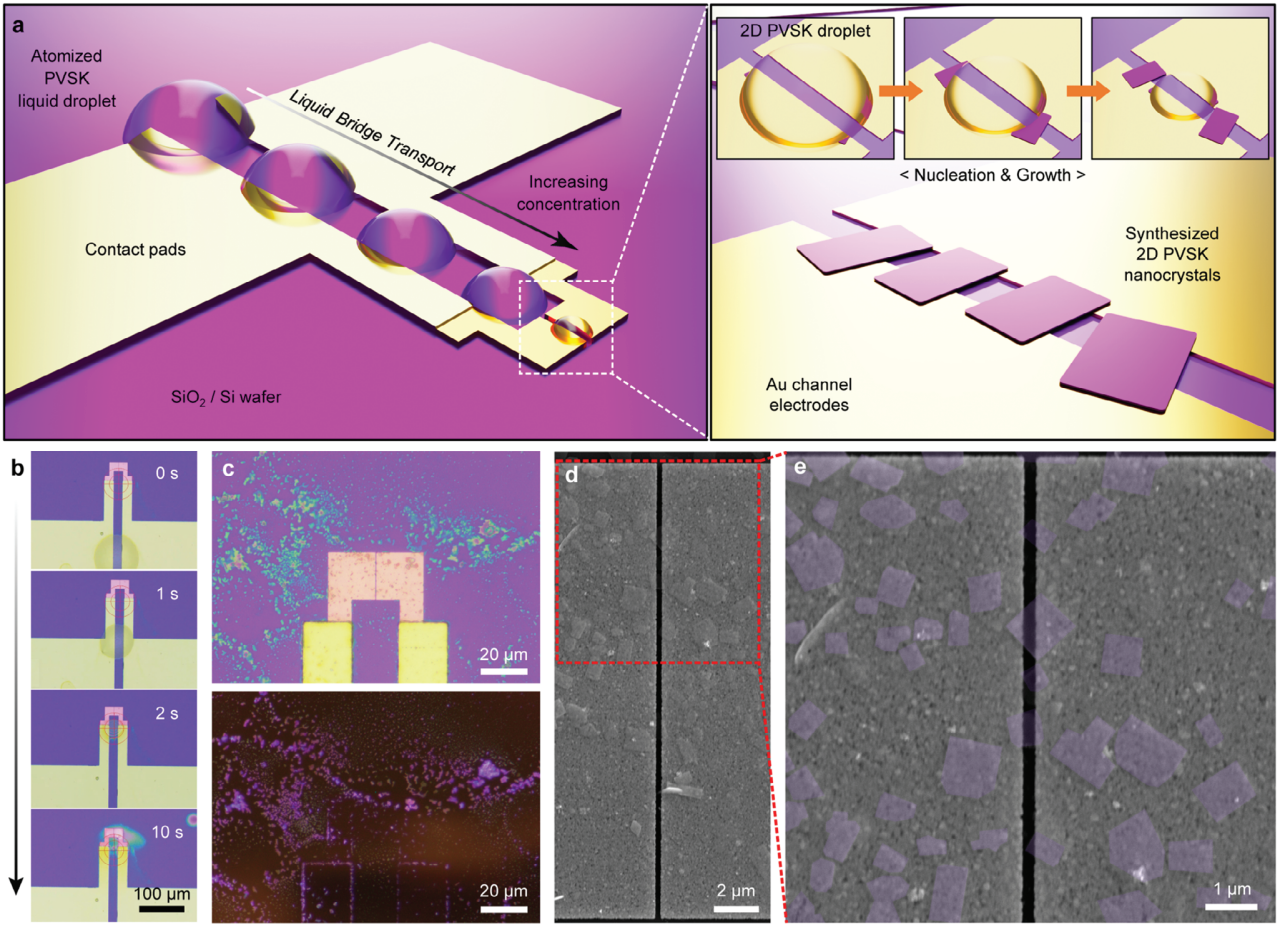
Liquid bridge effect 2D PVSK precursor droplet between the electrodes. a) Schematic illustration of liquid bridge effect‐based droplet pinning and transport for 2D PVSK precursor droplet between electrodes with enlarged schematic illustration of synthesized 2D PVSK single nanocrystals synthesized on top of and between two electrodes via the USSC process. b) Time‐lapse OM images of 2D PVSK precursor droplet transport on the electrodes. c) OM (top) and PL‐OM (bottom) images of (PEA)_2_PbBr_4_ single nanocrystals on the electrodes. d) SEM and e) enlarged SEM images of (PEA)_2_PbBr_4_ single nanocrystals on electrodes.

**Figure** **3**a shows a schematic illustration of the liquid bridge transport of the 2D PVSK precursor droplet on the substrate with electrodes and contact pads. The PVSK solution droplet coated within the micrometer‐sized gap between the contact pads became confined owing to the liquid bridge effect with different surface energies of the hexamethyldisilizane (HMDS) surface‐treated Si/SiO_2_ substrate and Au electrodes (Figure S21, Supporting Information). Then, as the solvent evaporates, the confined droplets move along the gap between the contact pads toward the electrodes and are located at the electrodes channels due to the liquid bridge transport effect.^[^ ^29^ ^]^ As a result, crystals nucleate near the contact line of the substrate‐droplet‐air interface, leading to the droplet‐pinned crystallization effect, primarily resulting in crystal growth near the electrode's edge.^[^ ^8^, ^30^, ^31^, ^32^ ^]^ The transportation of the PVSK solution droplet toward the electrodes is shown in Figure 3b and Video S1 (Supporting Information).

Consequently, this greatly enhances the probability of the synthesized crystals positioning themselves between the electrodes. Figures 3c and S22 (Supporting Information), show the OM and PL‐OM images, revealing that the edge areas of the electrode and contact pad have a higher concentration of crystals due to the droplet‐pinned crystallization effect, resulting in a much brighter purple‐blue PL. Figure 3d shows the SEM images of the electrode, revealing the morphology of the synthesized crystals between and on the electrodes. The enlarged SEM image shows well‐defined rectangular‐shaped synthesized crystals that formed between the electrodes (Figures 3e and S23, Supporting Information). The crystals formed between the electrodes are probably mostly suspended between them and may have bent slightly downward over the gap (Figure S24 and Note S1, Supporting Information).

### 2.3 2D PVSK Nanocrystal Photodetectors

**Figure 4:**
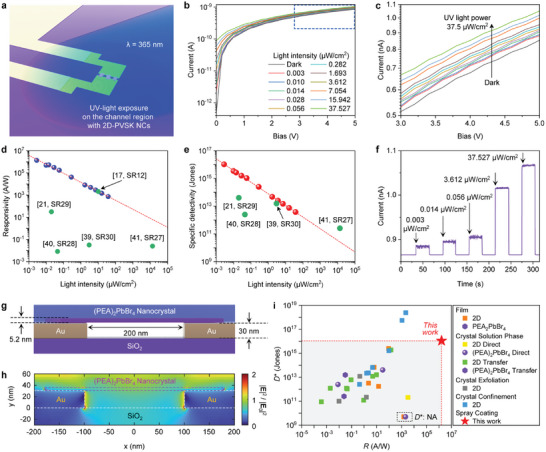
Characterization of (PEA)_2_PbBr_4_ nanocrystal UV PDs. a) Schematic diagram of (PEA)_2_PbBr_4_ nanocrystal PDs exposed to UV light source (*λ* = 365 nm). b) *I*‐*–V* and c) enlarged *I–*‐*V* characteristics of (PEA)_2_PbBr_4_ nanocrystal PDs under various intensities of UV light illumination and dark conditions. d) *R*, and e) *D* ^*^ of (PEA)_2_PbBr_4_ nanocrystal PDs under various intensities of UV light (5 V bias) with performance parameter values of previously reported PDs based on (PEA)_2_PbBr_4_ materials and (BA)_2_PbBr_4_^[^ ^12^ ^]^ indicated. f) Photoswitching characteristics of (PEA)_2_PbBr_4_ nanocrystal PDs under various intensities of UV light and dark conditions (5 V bias). g) Schematic image of the (PEA)_2_PbBr_4_ nanocrystal PDs device structure under UV light. h) Electric field intensity distribution for (PEA)_2_PbBr_4_ nanocrystal PDs under 365 nm light illumination in the cross‐sectional view. i) Comparison of the reported 2D perovskite material‐based PDs with those in our work with respect to *D* ^*^ and *R*. Related references are provided in Supplementary references 1–30. Related references are provided in Table S3, Supporting Information (denote “NA”: not available).

**Figure** **4**a schematically illustrates the UV‐PD device structures composed of Si/SiO_2_ substrate, Au electrodes, and 2D PVSK nanocrystals. A photograph showing the measurement of the device characteristics is shown in Figure S25 (Supporting Information). The photosensitivity of the UV‐PDs was estimated by measuring the current–voltage (*I*–*V*) characteristics under UV (365 nm) light with various light intensities. Figure 4b,c shows the *I*–*V* and enlarged *I*–*V* characteristics of the photodetectors, respectively, where the current increases gradually with increasing illumination power of UV light owing to the increased number of incident photons.

To quantitatively compare the performances of the different types of PVSK PDs, *R* was calculated using the following equations:^[^ ^33^ ^]^

(1) $$R=\frac{I_{\mathrm{light}}-I_{\mathrm{dark}}}{P_{\mathrm{inc}}}$$

where *I*_light_ is the current under light illumination, *I*_dark_ is the current under dark condition, and *P*_inc_ is the incident illumination power. In addition, D^*^ is a key performance parameter for PDs, typically indicating the smallest detectable signal, as follows:^[^ ^4^, ^34^ ^]^

(2) $$D^{*}=\frac{{\left(AB\right)}^{1/2}}{NEP}\;\left(\mathrm{cm}\;Hz^{1/2}W^{-1}\right)$$

(3) $$NEP=\frac{{\bar{{i_{n}}^{2}}}^{1/2}}{R}\;\left(W\right)$$

where A is the effective area of the detector in cm^2^, B is the bandwidth, NEP is the noise equivalent power, and ${\bar{{i_{n}}^{2}}}^{1/2}$ is the measured noise current. If shot noise from the dark current is the major factor contributing to noise limiting the D^*^, then D^*^ can be simplified as.^[^ ^34^, ^35^ ^]^

(4) $$D^{*}=\frac{R}{{\left(2e\cdot I_{\mathrm{dark}}/A\right)}^{1/2}}$$

The *R* and *D* ^*^ values of PDs under UV light at various intensities are shown in Figure 4d,e, along with the reported performance metrics of UV PDs based on (PEA)_2_PbBr_4_ crystals highlighted in the graphs. These *R* and *D* ^*^ are critical parameters that indicate how sensitively PDs can detect light and how accurately they can capture signals, playing a key role not only in evaluating device performance but also in identifying errors and finding solutions for improvement.^[^ ^4^, ^36^ ^]^ The enhancements in *R* and *D* ^*^ significantly increased as the light intensity decreased, which is a commonly observed phenomenon.^[^ ^37^, ^38^ ^]^

Under a UV light intensity of 0.003 µW cm^−2^, our (PEA)_2_PbBr_4_ single nanocrystal‐based PDs achieved remarkably ultra‐high *R* and *D* ^*^ values of 1.6 × 10^6^ A W^−1^ and 1.1 × 10^16^ Jones, respectively, which are greatly superior to the performance metrics reported in other (PEA)_2_PbBr_4_ based studies.^[^ ^21^, ^39^, ^40^, ^41^ ^]^ Notably, under 10 µW cm^−2^ UV light, our photodetectors (with a 200 nm electrode gap) achieved *R* values comparable to those of high‐performance butylammonium (BA)‐based (BA)_2_PbBr_4_ single‐crystal photodetectors with a narrow 100 nm electrode gap using monolayer graphene electrodes for effective electrical contact but significantly higher D^*^.^[^ ^17^ ^]^ Moreover, the on/off photoswitching characteristics of the photodetectors under various UV light intensities exhibit stable output signals across repeated on/off cycles, demonstrating the reliable operation of our sensors (Figure 4f). The rise time (*t*_r_, defined as the time required to go from 10% to 90% of the maximum photocurrent) and decay time (*t*_d_, defined as the time required to go from 90% to 10% of the maximum photocurrent) of our devices were estimated shorter than 22.1 ms, as shown in Figure S26 (Supporting Information).

This underscores the fact that such results can be realized with a straightforward and effective USSC technique, likely due to the small, thin crystals adhering well to the morphology of the bottom contact gold electrodes. One factor contributing to the high performance of our PDs is the use of 200 nm narrow‐gap electrodes, which enable more effective capture of charge carriers generated in the PVSK crystal compared to electrodes with micrometer‐scale gaps. Moreover, the USSC approach, which enables the fabrication of PDs with bottom contact electrodes, may contribute to the high performance of the PDs by helping to prevent additional damage to the single crystal during the metal electrode fabrication process, a common drawback of top contact electrode‐based devices.^[^ ^42^, ^43^, ^44^ ^]^ Additionally, the plasmonic gap mode induced by gold electrodes with nanometer‐scale gaps and the lightning rod effect caused by the gold surface roughness can enhance light scattering and amplification, potentially increasing the photocurrent of the photodetectors. To theoretically explore the localized surface plasmon resonance (LSPR) and scattering effects of gold electrodes with a 200‐nm pitch in the photodetector system (Figure 4g), the electric field intensity distribution (i.e., |*E*(*ω*)|^2^/*E*_0_^2^) was numerically calculated (Figure 4h). Under the illumination of UV light with 365 nm wavelength, the electric field distribution is amplified around two times in the regions surrounding the nanocrystals and the electrode edges. In this analysis, smooth gold surfaces were assumed for simpler calculations. Gold surfaces are much rougher in reality (Figure 3e) and would show a stronger light amplification effect of the lightning rod effect within (PEA)_2_PbBr_4_ nanocrystals. It is noteworthy that our 2D PVSK PDs demonstrated the highest *R* and *D* ^*^ values compared with those previously reported not only among 2D PVSK PDs but also among all PVSK PDs (Figure 4i; Table S3, Supporting Information). The observed electrical readouts surpassed the state‐of‐the‐art for 2D perovskite materials, demonstrating excellent *R*−*D* ^*^ correlation. This represents *R* values 486 times higher than the best performance previously reported for 2D PVSK PDs. Notably, our excellent *R*−*D* ^*^ correlation is highly competitive with previously reported 3D PVSK PDs (Figure S27 and Table S4, Supporting Information).

### 2.4 Wafer Scale PDs Array and Pattern Recognition

**Figure 5:**
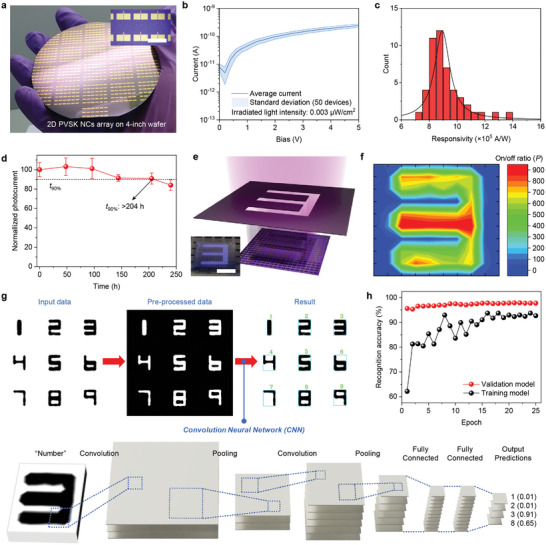
Characterization of large area 2D PVSK PD arrays for smart pattern recognition. a) Photograph of the (PEA)_2_PbBr_4_ nanocrystal PD array on a 4‐inch Si/SiO_2_ wafer (inset: OM images of PD array). b) *I‐V* curves of the 50 PDs from the PD array on a 4‐inch Si/SiO_2_ wafer. Lines and shaded areas are mean ± standard deviation for 50 PDs (λ = 365 nm, intensity 0.003 µW cm^−2^). c) *R*‐value distribution of the 50 PDs from the PD array on a 4‐inch Si/SiO_2_ wafer (5 V bias). d) Thermal stability tests of the PDs in N_2_ conditions at a temperature of 90 °C (λ = 365 nm, intensity 37.5 µW cm^−2^). e) Schematic diagram of 19 × 19 (PEA)_2_PbBr_4_ nanocrystal PD array partially exposed to UV light in the shape of the number ‘3’ (λ = 365 nm, intensity 37.5 µW cm^−2^) (inset: photograph image of 19 × 19 PD array under number ‘3’‐shaped light illumination). f) Spatial photosensing mapping of the PD arrays under UV light illumination in the shape of the number ‘3′. g) Schematic illustrating the CNN structure for number shape pattern recognition based on this device. h) Recognition accuracy of the number of pattern shapes for training and validation models.

To explore the potential of our USSC approaches for large‐area applications, leveraging its capability for extensive coverage, we fabricated 4‐inch wafer‐scale PD arrays by applying the USSC of 2D PVSK precursor solution to a 4‐inch wafer with a pre‐deposited electrode array (**Figure** **5**a). Video S2 (Supporting Information), shows the USSC process, in which the nozzle moves across the 4‐inch wafer to uniformly apply the 2D PVSK precursor solution. Figure S28 (Supporting Information), presents a collection of OM images for each PD in the PD array. These OM images confirm that the crystals are well positioned between the electrodes. We selected 50 PDs to further analyze the performance uniformity of the PD arrays (Figure S29, Supporting Information) The 50 samples had nearly identical *I‐V* curve characteristics and *R* values (Figure 5b,c, respectively), suggesting good uniformity of the PD performances over a large scale, while a few devices showed a slight decrease in current at 0.2 V bias. The average *R* value of the 50 samples was 8.9 × 10^5^ A W^−1^; the highest *R* value was 1.4 × 10^6^ A W^−1^, with a narrow standard deviation (SDs) of 14%. Individual *I‐*–*V* curves of these 50 PDs are also shown in (Figure S30, Supporting Information). We further conducted thermal stability tests on our PDs at 90 °C in a N_2_‐filled glovebox (Figure 5d). The PDs maintained 90% of their initial photocurrent for more than 200 h. The working thermal stability value at this level is competitive with that compared of other lateral‐type perovskite photodetectors (PDs),^[^ ^45^, ^46^, ^47^, ^48^ ^]^ while showing lower stability for humidity and continuous light exposure, with device performance significantly degrading within several hours and tens of hours, respectively, probably due to the lateral‐type device structure exposing the semiconductor to air and the high surface area resulting from the nano‐sized crystals (Figure S31, Supporting Information).

Moreover, the photo‐imaging sensing performance of the 19 × 19 PD array was also investigated by exposing it to UV light in the shape of numbers 0–9 at the center of the sensor matrix (Figures 5e and S32a, Supporting Information). The photon signals are described by 2D mapping as a function of the photocurrent‐to‐dark current ratio (Figures 5f and S32b, Supporting Information), clearly indicating that the distribution of each result matches the exposed light patterns corresponding to the number shapes.

To implement an automatic number pattern recognition system on our computing platform, we built a TensorFlow platform‐based convolutional neural network (CNN) architecture tailored to this device (Figure 5g). We trained the CNN using an modified MNIST dataset of 60000 samples based on the number shapes (0 to 9) obtained. Figure 5h demonstrates that the network achieved a pattern recognition accuracy of over 95%, which is notably high enough to ensure clear distinction even with a relatively low‐accuracy neural network, comparable to the performance under ideal conditions, and significantly superior to the accuracy obtained with a conventional test program model. These results underscore the potential of 2D perovskite nanocrystal PD arrays for the development of robust automatic recognition systems with powerful pattern recognition capabilities.^[^ ^49^, ^50^, ^51^, ^52^ ^]^

## 3 Conclusion

In this study, we have developed a scalable synthetic approach to fabricate 2D PVSK single nanocrystals over a large area using the USSC method and their applications for high‐performance large‐area PDs. The atomized precursor solution from the USSC process enabled the synthesis of purple‐blue 2D PVSK single crystals with tunable sizes ranging from tens to hundreds of nanometers in a uniform and scalable manner. This approach also proved versatile, allowing for the synthesis of red and green 2D PVSK single crystals. Moreover, by employing liquid bridge transport, which promotes dominant crystal synthesis between electrodes, we achieved a remarkable 100% device performance yield across a 4‐inch wafer scale. This process facilitated the fabrication of a PD array with outstanding performance, achieving a *R* of 1.6 × 10^6^ A W^−1^ and a *D* ^*^ of 1.1 × 10^16^ Jones. Furthermore, leveraging the high‐resolution photomapping capabilities of the PD arrays, we successfully demonstrated a smart vision system for automatic shape recognition using CNN implementation. The insights in this study offer guidance for the mass production of nanoscale 2D PVSK single nanocrystals, the realization of large‐area electronic arrays, and the design of advanced image detection systems.

## 4 Experimental Section

### Materials

Anhydrous chlorobenzene (CB), dimethylformamide (DMF), acetonitrile (AN), dichlorobenzene (DCB), and acetone were purchased commercially (Sigma‐Aldrich). Lead bromide (PbBr_2_), lead iodide (PbI_2_), and tin iodide (SnI_2_) were purchased commercially (Sigma‐Aldrich). PEABr and PEAI were purchased from (GreatCell Solar). 950 K polymethyl methacrylate (PMMA) e‐beam resist, e‐beam resist developer, AZ 1518 photoresist, and MF26A photoresist developer were purchased commercially (MicroChemicals). All of these materials were used as received without further purification.

### Synthesis and Characterization of 2D PVSK Crystals by USSC

10 µmol of MX_2_ (M = Pb or Sn, X = Br or I) and 20 µmol of L·HX (L = PEA, X = Br or I) were dissolved in 2 mL of DMF/CB cosolvent (1:1 volume ratio) and stored at room temperature in an N_2_‐filled glovebox. The concentrated stock solutions were then diluted using a solvent system with a CB/AN/DCB cosolvent. For (PEA)_2_PbBr_4_, (PEA)_2_PbI_4_, (PEA)_2_SnI_4_, the stock solution was with a CB/AN/DCB (volume ratio of Stock:CB:AN:DCB = 85:7300:2900:8.5/20:1050:300:1/30:600:900:1, respectively.) An automated ultrasonic spray coating system (Sono‐Tek, USA) was utilized to fabricate 2D perovskite crystal films using the prepared precursor solutions for the three different 2D crystals. The nozzle‐to‐substrate distance was maintained at 50 mm. The ink flow rate and number of coating cycles were adjustable, ranging from 0.1 to 1.0 mL min^−1^, depending on the desired 2D perovskite crystal features and density. The processing temperature of the coating stage was maintained at 70 °C. Nitrogen gas was used to control the shaping air pressure, the coating speed was set to 60 mm s^−1^, and the ultrasonic spray nozzle (120 kHz) was moved in an arc‐shaped pattern. Subsequently, the substrate was annealed at 70 °C for 10 min. For the conventional solvent drying, 10 µL of 2D perovskite precursor solution was dropped onto the substrate and annealed at 70 °C for 10 min. (detailed information on USSC) For the spin coating method, 20 µL of 2D perovskite precursor solution was dropped onto the Si/SiO_2_ substrate, spin‐coated at 2000 rpm for 60 s, and then annealed at 70 °C for 10 min.

Bright‐field optical images were collected using a custom microscope (Olympus BX53). The samples were excited with a light source (012‐63000; X‐CITE 120 REPL LAMP). Photoluminescence spectra were obtained using a spectrometer (SpectraPro HRS‐300). SEM images were obtained using a Hitachi S‐4800 cold SEM microscope. The crystalline structures were examined using X‐ray diffraction (XRD, PANalytical Empyrean). The AFM height image results were obtained using a Bruker iCON Dimension system in the tapping mode for soft topography. TEM electron diffraction patterns and morphological images were acquired using a Gatan US1000XP CCD camera equipped on a 200 kV Tecnai Osiris.

### Fabrication and Characterization of 2D PVSK Crystal Photosensor Array

Au electrodes were fabricated on Si/SiO_2_ substrates using conventional e‐beam lithography (JEOL JBX‐8100FS), photolithography (Heidelberg MLA150 Maskless Aligner), and thermal evaporation (CHA E‐Beam Evaporator). First, the HMDS solution was spin‐coated on Si/SiO_2_ wafers. After patterning the e‐beam resist, Ti/Au (3/30 nm) was thermally evaporated onto the substrate, followed by acetone cleaning to remove the e‐beam resist to prepare electrodes and align the key. Then, after the photoresist was patterned on the substrate, Ti/Au (3/40 nm) was thermally evaporated onto the substrate again, followed by acetone cleaning to remove the photoresist for contact pad preparation. Finally, the USS coating and annealing process were performed using the 2D perovskite precursor solution on the fabricated substrate with electrodes and contact pads. The current–voltage characteristics and photo‐response of the photosensors were measured in a vacuum chamber using a 4200‐SCS semiconductor parametric analyzer with a 365 nm UV lamp. The photoactive effective area was determined using SEM images.

### Finite‐Difference Time‐Domain (FDTD) Simulation Methods

The electric field distribution was calculated using a commercial FDTD solver (Lumerical Solutions). The simulation was performed for a 2D system in the x‐ and z‐directions. A periodic boundary condition in the x‐direction was used with a sufficiently large periodicity (10 µm) to remove the unwanted effect from artificial periodicity. A perfectly matched layer in the z‐direction was used. The plane wave source was excited to emulate the incident light. The thickness of the perovskite active layer was 5.2 nm, and the maximum mesh size near the perovskite active layer was 0.25 nm to resolve the small thickness. The optical constant of gold was taken from the tabulated data^[^ ^53^ ^]^ and that of the perovskite from a previously reported study.^[^ ^54^ ^]^

### CNN Pattern Recognition

CNN was developed for handwritten digit classification, utilizing the MNIST dataset with TensorFlow and Keras. The dataset consists of 60 000 training and 10 000 test images. The CNN architecture included two convolutional layers (32 and 64 filters, ReLU activation), followed by max pooling and dropout layers to prevent overfitting. The output was flattened and passed through a dense layer with 128 units and a final dense layer with 10 units (softmax activation). The model was compiled with categorical crossentropy and the Adam optimizer. In addition, the model was trained for 25 epochs with a batch size of 128. For digit recognition, OpenCV was used to preprocess input images. Images were converted to grayscale, thresholded, and morphological transformations were applied to extract contours. Each contour was processed to isolate individual digits, which were resized to 28 × 28 pixels and normalized. Predictions were made using the trained model, and results were visualized on the original images.

## Conflict of Interest

The authors declare no conflict of interest.

## Author Contributions

Y.H.L. and W.‐J.L. contributed equally to this work. Y.H.L., W.‐J.L., and L.D. conceived the idea for the study. Y.H.L. and W.‐J.L. were responsible for synthesizing and characterizing the materials, as well as fabricating the detector array devices. G.S.L. contributed to additional material synthesis. J.Y.P., H.Y., S.‐D.B, G.K. characterized materials. J.M. performed plasmonic simulations and data analysis. B.Y. and T.J.P. conducted high‐resolution TEM analysis. Y.W. and J.H.O. carried out CNN‐based pattern recognition. H.L. was responsible for literature review and characterization tasks. Y.H.L., W.‐J.L., L.D., drafted the manuscript. J.M. provided support for the USSC system and instrumental characterizations, and L.D. guided all aspects of the project. All authors engaged in discussions regarding the experiments and contributed to writing the manuscript.

## Supporting information



Supporting Information

Supplemental Video1

Supplemental Video2

## Data Availability

The data that support the findings of this study are available from the corresponding author upon reasonable request.
