# Supplementary material for: Large‐Scale 2D Perovskite Nanocrystals Photodetector Array via Ultrasonic Spray Synthesis
Source: Adv Mater. 2025 Feb 19;37(12):2417761. doi: 10.1002/adma.202417761 (PMC11938026; doi:10.1002/adma.202417761)
Supplement: Supplementary file 1 — Supporting Information [file ADMA-37-2417761-s003.pdf]

ADVANCED MATERIALS

Supporting Information

for *Adv. Mater.*, DOI 10.1002/adma.202417761

Large-Scale 2D Perovskite Nanocrystals Photodetector Array via Ultrasonic Spray Synthesis

Yoon Ho Lee, Won-June Lee, Gang San Lee, Jee Yung Park, Biao Yuan, Yousang Won, Jungho Mun, Hanjun Yang, Sung-Doo Baek, Haeun Lee, Joon Hak Oh, Timothy J. Pennycook, Gwangwoo Kim, Jianguo Mei and Letian Dou**

Supporting Information

Large-Scale 2D Perovskite Nanocrystals Photodetector Array via Ultrasonic Spray Synthesis

Yoon Ho Lee[1,2,3,†], Won-June Lee[2,†,*], Gang San Lee[4], Jee Yung Park[1], Biao Yuan[5], Yousang Won[6], Jungho Mun[4,7], Hanjun Yang[1,2], Sung-Doo Baek[1], Haeun Lee[3], Joon Hak Oh[6], Timothy J. Pennycook[5], Gwangwoo Kim[8], Jianguo Mei[2], Letian Dou[1,*]

[1] Davidson School of Chemical Engineering, Purdue University, West Lafayette, IN 47907, USA

[2] James Tarpo Jr. and Magaret Tarpo Department of Chemistry, Purdue University, West Lafayette, IN 47907, USA

[3] Department of Materials Science and Engineering, Sungshin Women's University, Seoul 01133, Republic of Korea

[4] Elmore Family School of Electrical and Computer Engineering, Purdue University, West Lafayette, IN 47907, USA.

[5] EMAT, University of Antwerp, Groenenborgerlaan 171, 2020 Antwerp, Belgium

[6] School of Chemical and Biological Engineering, Institute of Chemical Processes, Seoul National University, Seoul 08826, Republic of Korea

[7] POSCO-POSTECH-RIST Convergence Research Center for Flat Optics and Metaphotonics, Pohang University of Science and Technology (POSTECH), Pohang 37673, Republic of Korea

[8] Department of Engineering Chemistry, Chungbuk National University, Cheongju, 28644, Republic of Korea

[†] These authors contributed equally: Yoon Ho Lee, Won-June Lee
* Corresponding authors: E-mail: dou10@purdue.edu and lee4458@purdue.edu

Table of Contents

Supplementary Figure S1-S32

Supplementary Table S1-S4

Supplementary Video S1 & S2

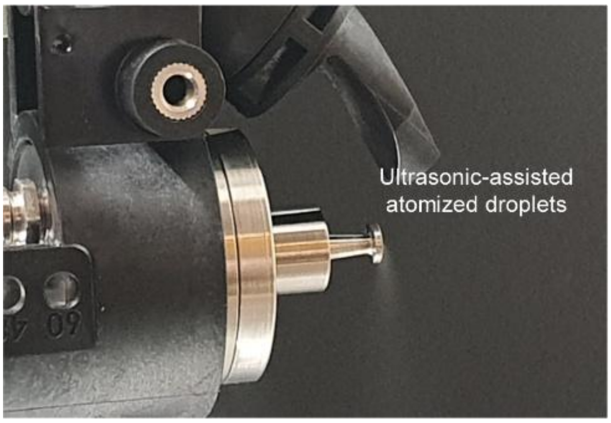

Figure S1. Photograph image of spray nozzle with atomized $(PEA)_2PbBr_4$ precursor solution droplets during USSC process

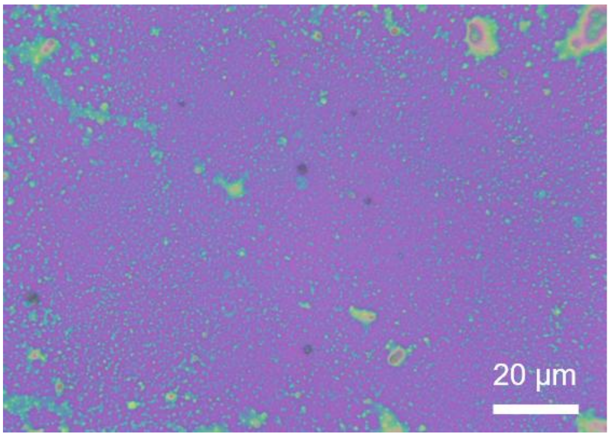

Figure S2. Corresponding OM image of $(PEA)_2PbBr_4$ 2D perovskite crystals synthesized by USSC.

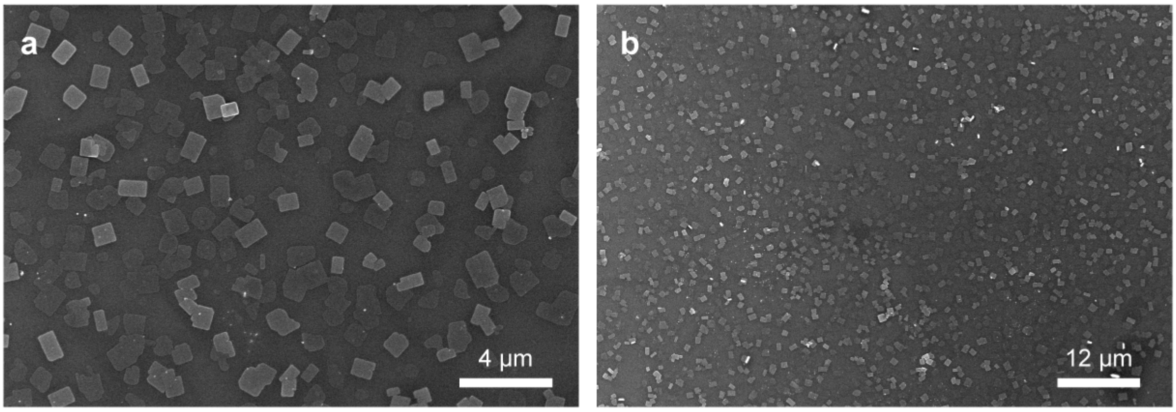

Figure S3. SEM images on $(PEA)_2PbBr_4$ single nanocrystals on SiO_2 wafer synthesized by USSC for 0.5 ml/min dispensing rate condition.

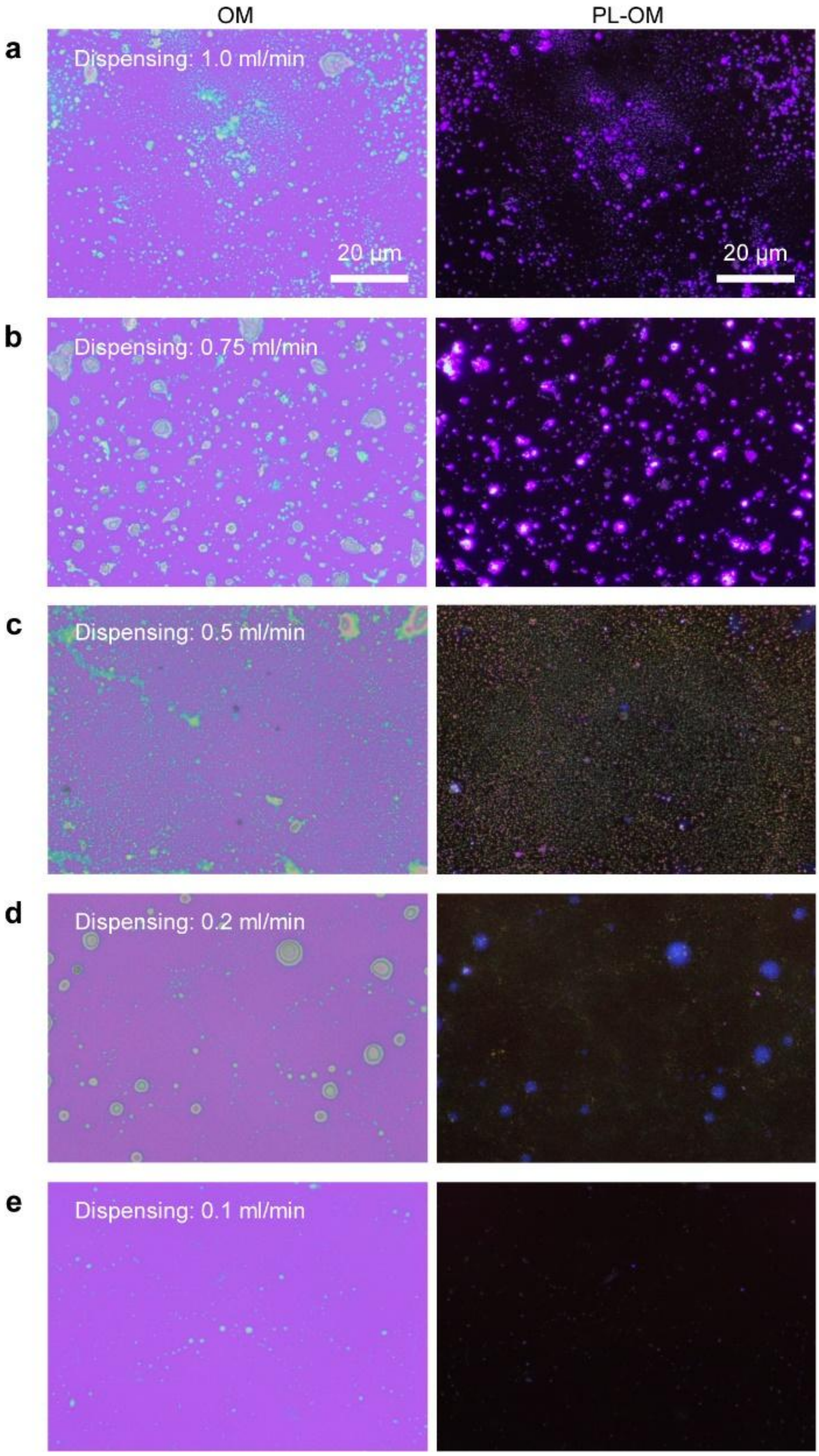

Figure S4. OM (left) and PL-OM (right) images of $(PEA)_2PbBr_4$ crystals under various dispensing rate USSC conditions including a) 1.0, b) 0.75, c) 0.5, d) 0.2, and e) 0.1 ml/min.

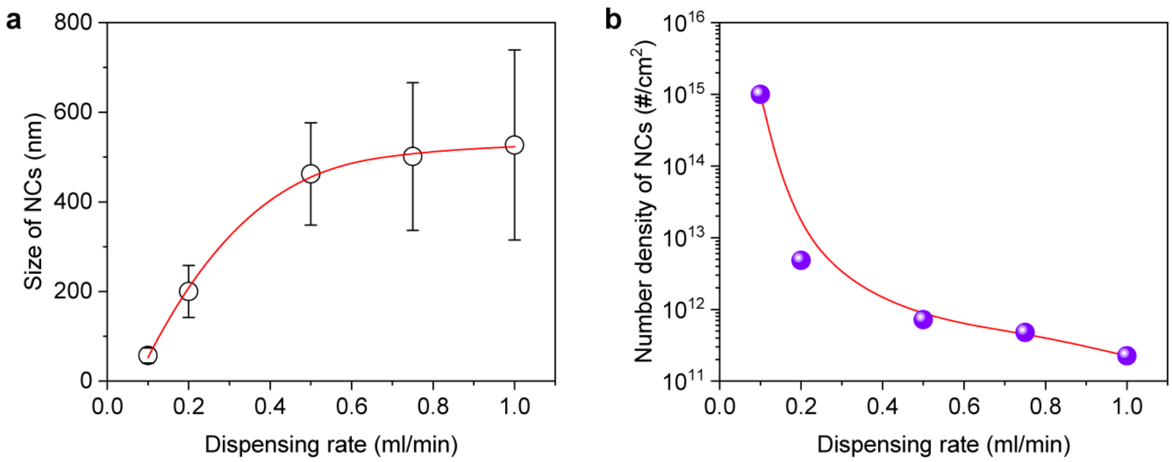

Figure S5. a) sizes and b) number densities of $(PEA)_2PbBr_4$ crystals under various dispensing rate USSC conditions.

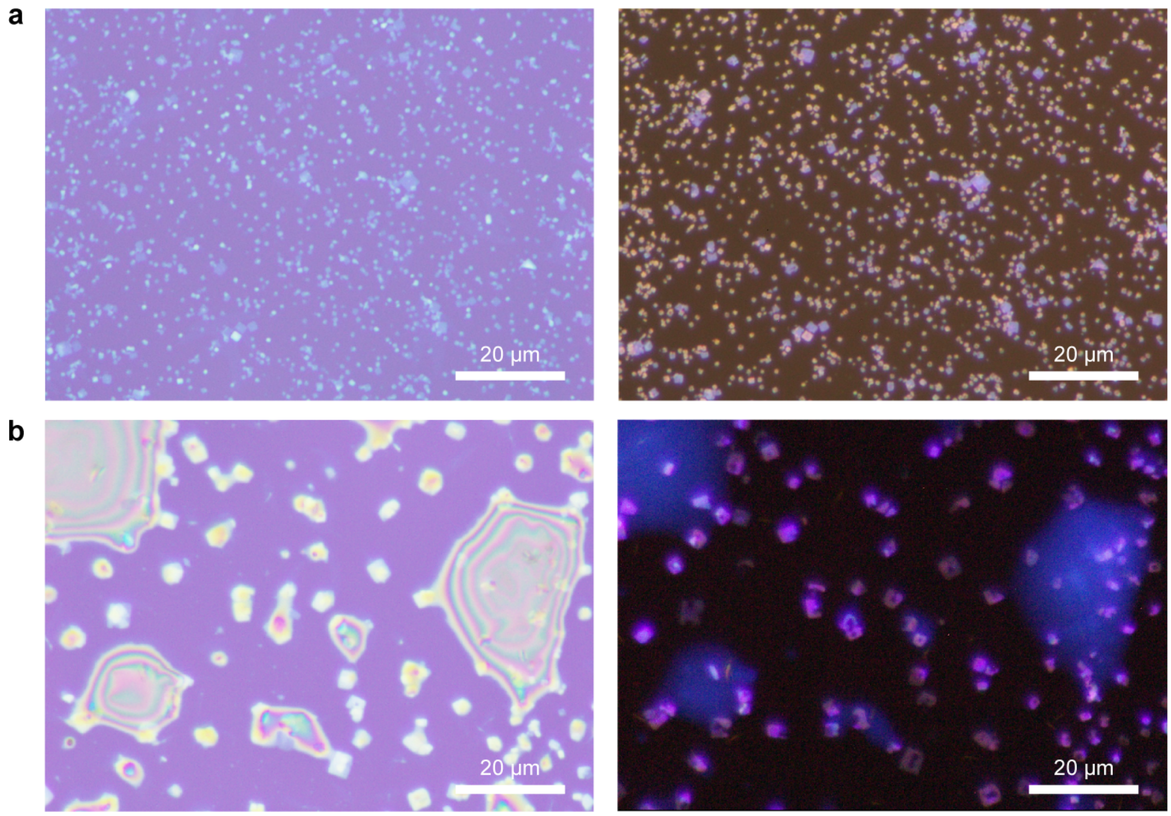

Figure S6. OM (left) and PL-OM (right) images of purple-blue-colored $(PEA)_2PbBr_4$ nanocrystals synthesized by a) USSC and b) conventional solvent drying method.

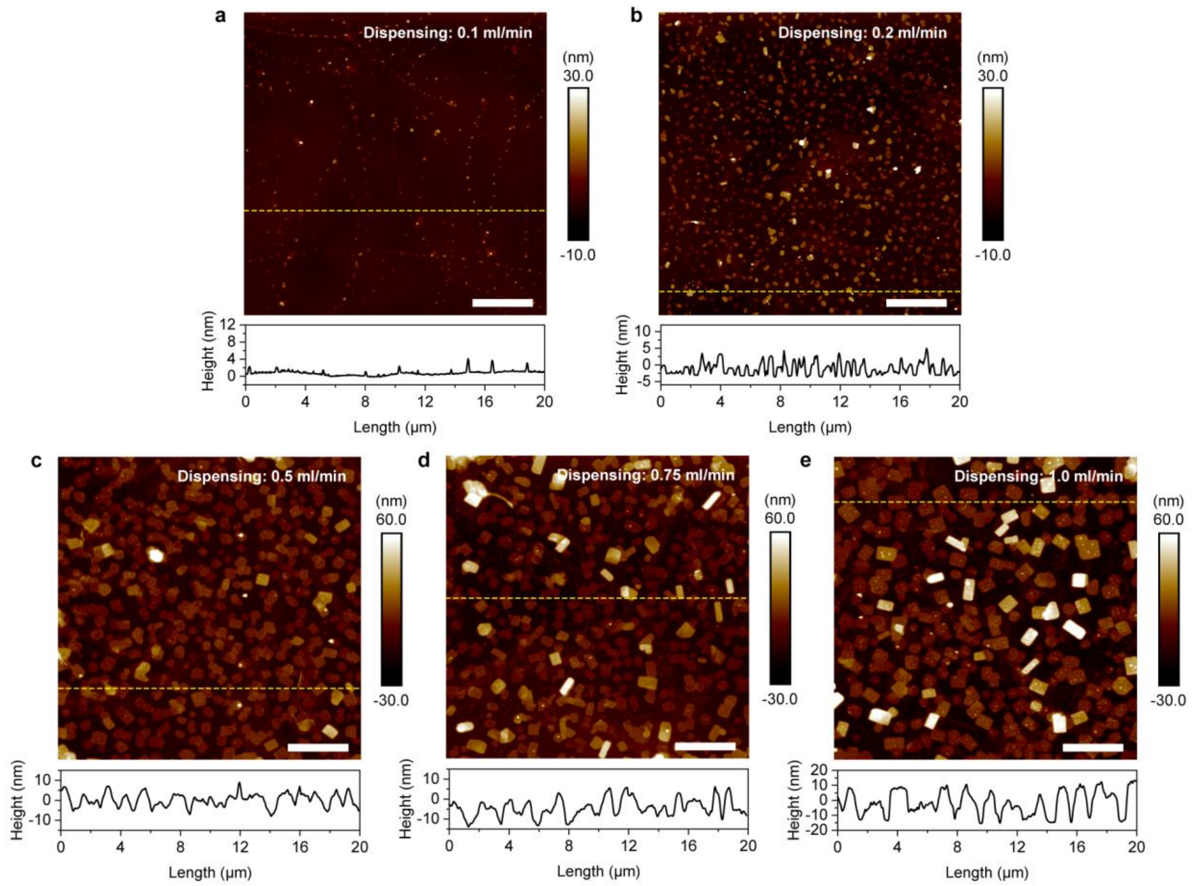

Figure S7. AFM height images of $(PEA)_2PbBr_4$ crystals under various dispensing rates of USSC conditions including a) 0.1, b) 0.2, c) 0.5, d) 0.75, and e) 1.0 ml/min.

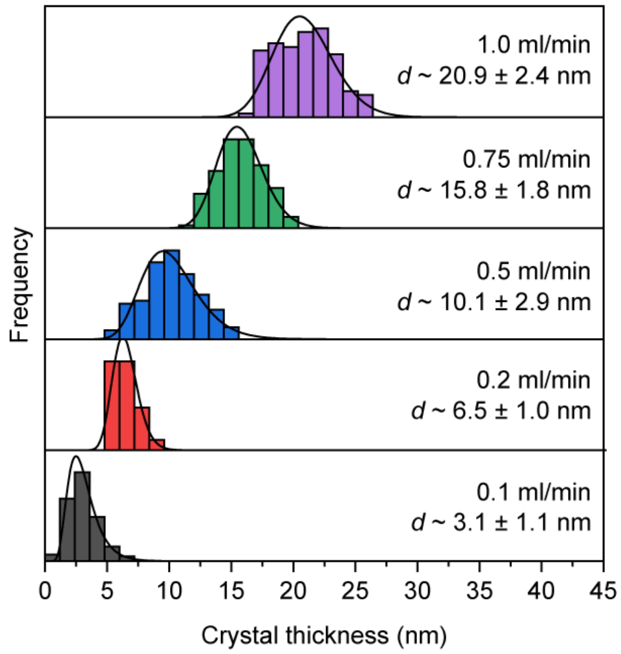

Figure S8. Statistical distribution of thickness of the synthesized $(PEA)_2PbBr_4$ crystals under various dispensing flow rates

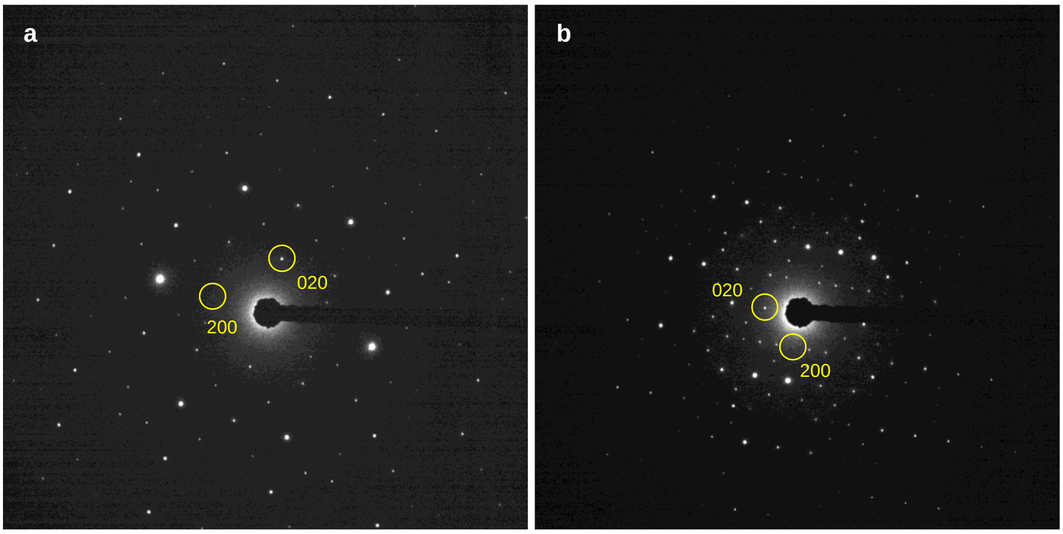

Figure S9. SAED pattern of $(PEA)_2PbBr_4$ crystals synthesized by USSC.

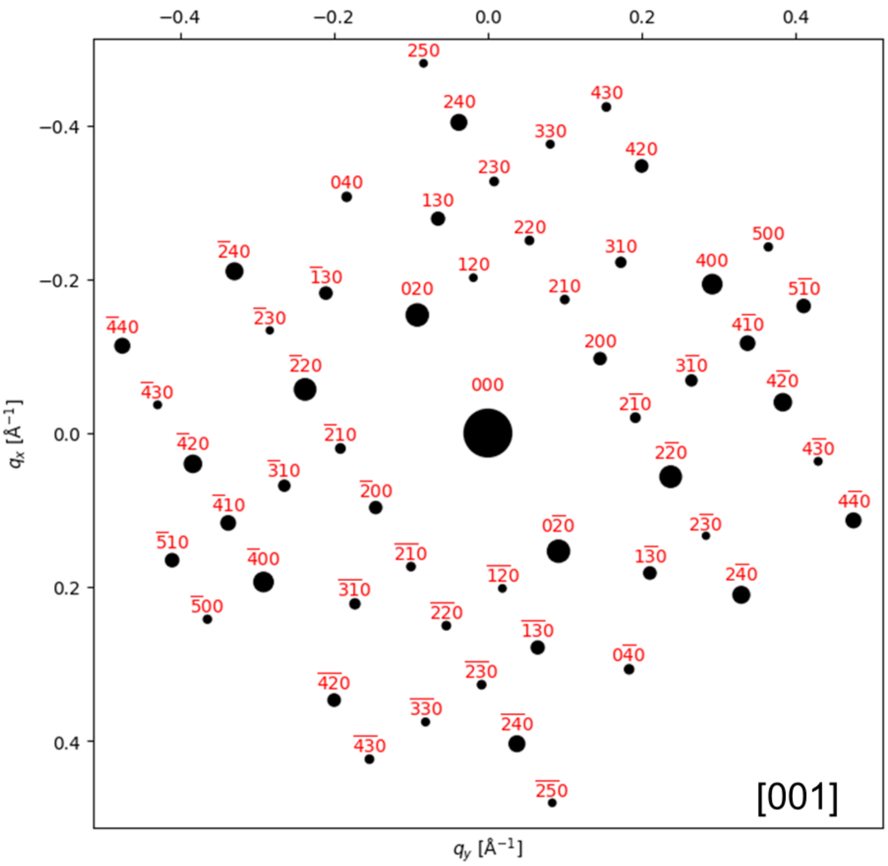

Figure S10. Simulated dynamical electron diffraction of $(PEA)_2PbBr_4$ synthesized by USSC.

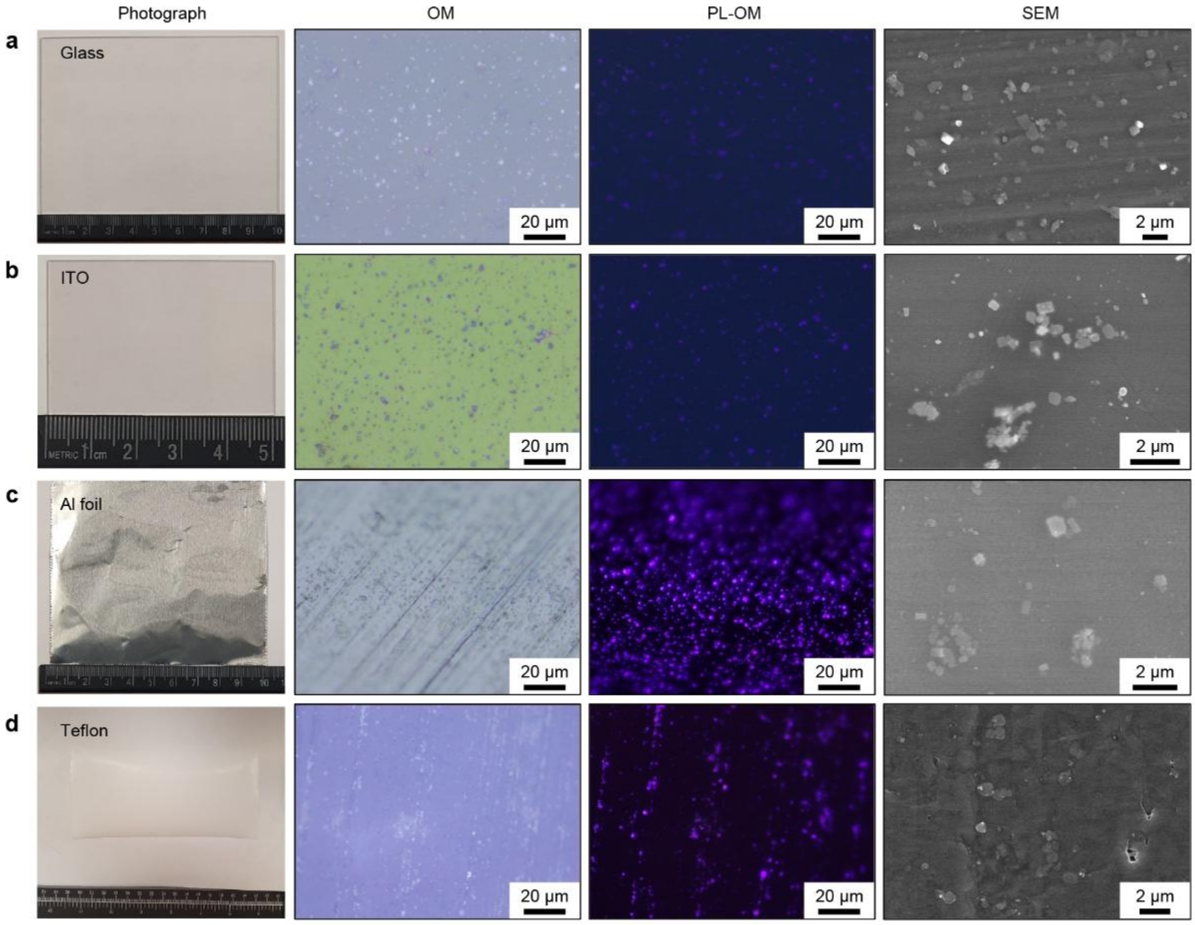

Figure S11. Photograph (left), OM (middle-left), PL-OM (middle-right), and SEM (right) images of $(PEA)_2PbBr_4$ nanocrystals based on USSC process on a) glass, b) ITO, c) Al foil, and d) Teflon sheet. (All substrates were not subjected to any additional hydrophilic treatment.)

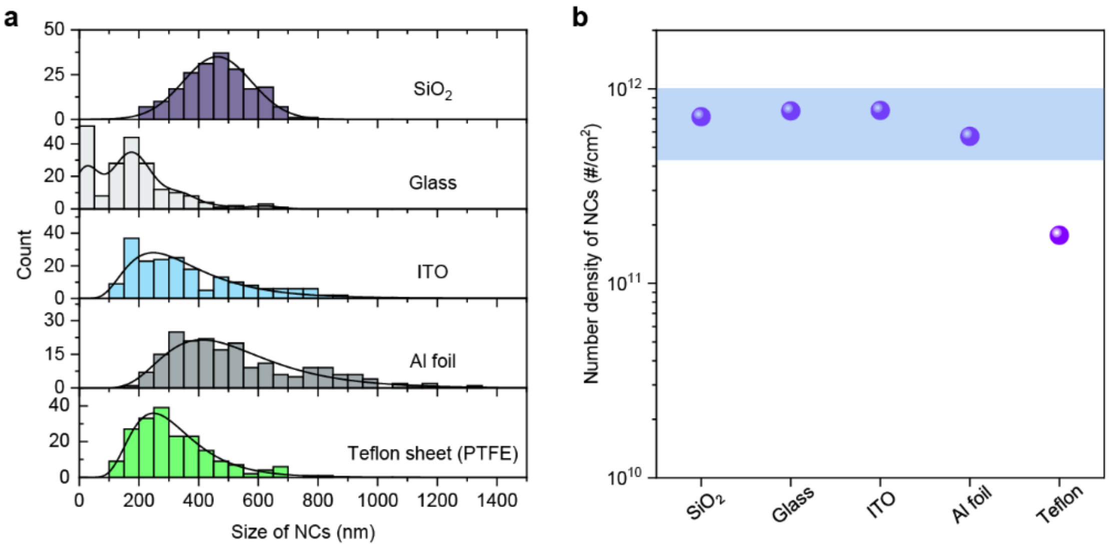

Figure S12. a) Statistical size distributions and b) number densities of $(PEA)_2PbBr_4$ nanocrystals based on USSC process on various types of substrates.

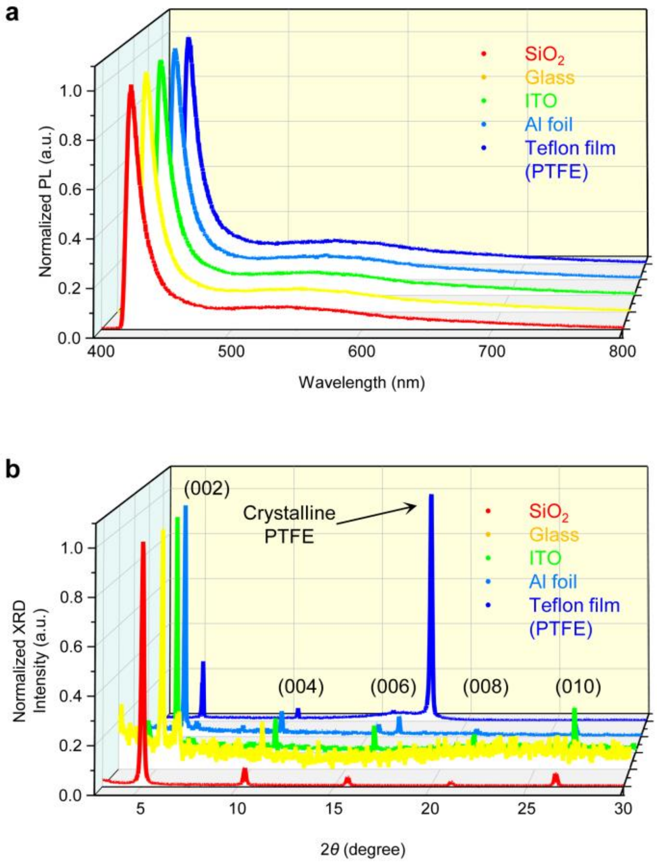

Figure S13. a) PL and b) XRD spectra of $(PEA)_2PbBr_4$ nanocrystals based on USSC process on various types of substrates.

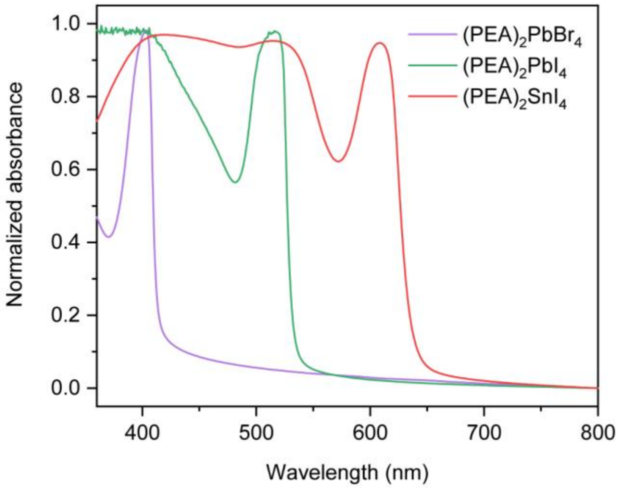

Figure S14. UV–visible absorption spectra of $(PEA)_2PbBr_4$, $(PEA)_2PbI_4$, and $(PEA)_2SnI_4$ 2D PVSK nanocrystals.

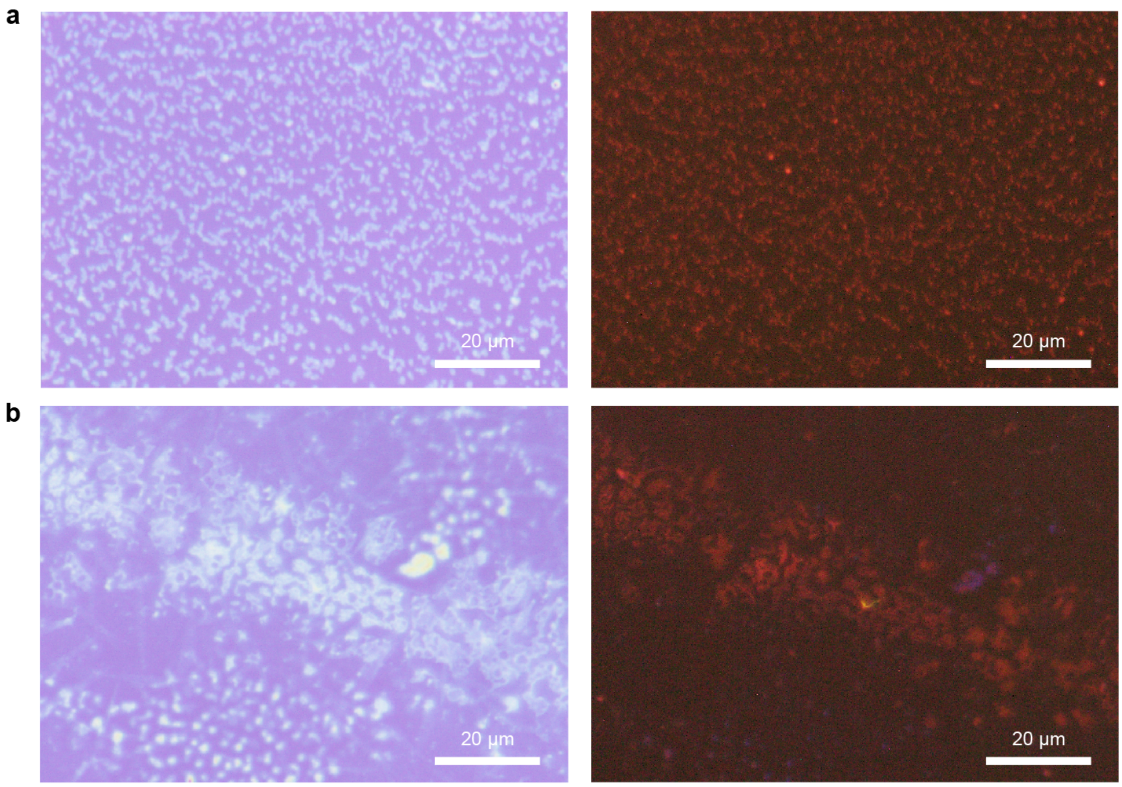

Figure S15. OM (left) and PL-OM (right) images of red-colored $(PEA)_2SnI_4$ nanocrystals synthesized by a) USSC and b) conventional solvent drying method.

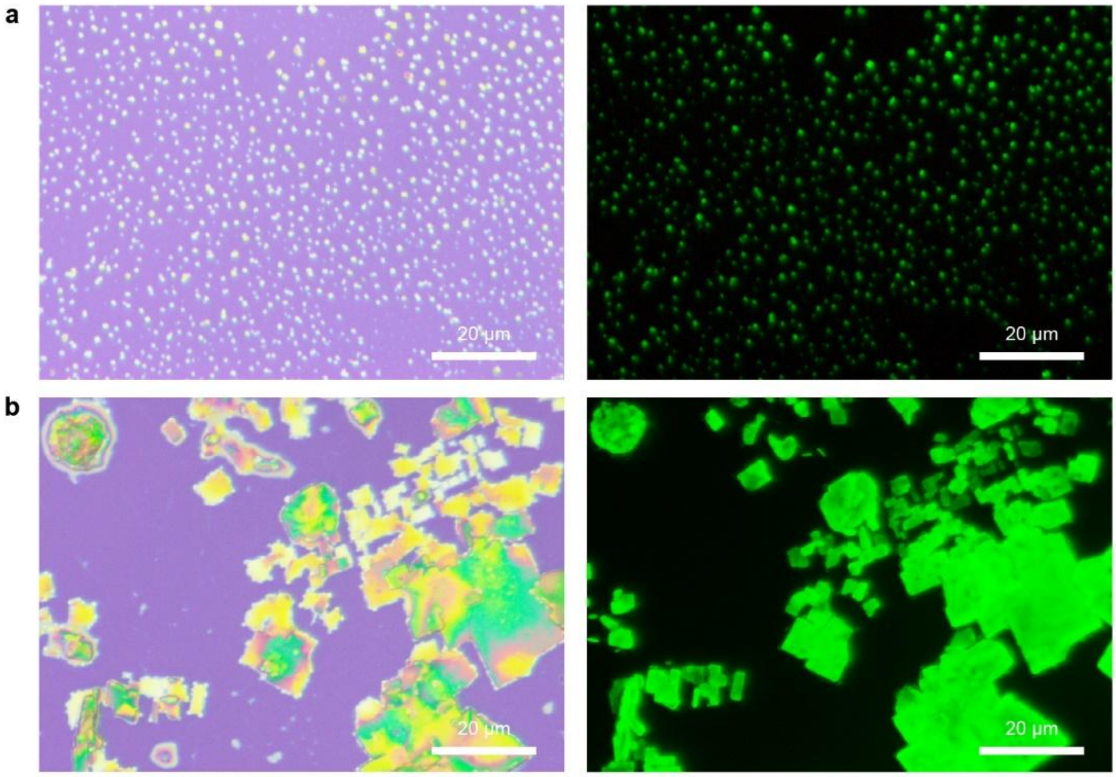

Figure S16. OM (left) and PL-OM (right) images of green-colored $(PEA)_2PbI_4$ nanocrystals synthesized by a) USSC and b) conventional solvent drying method.

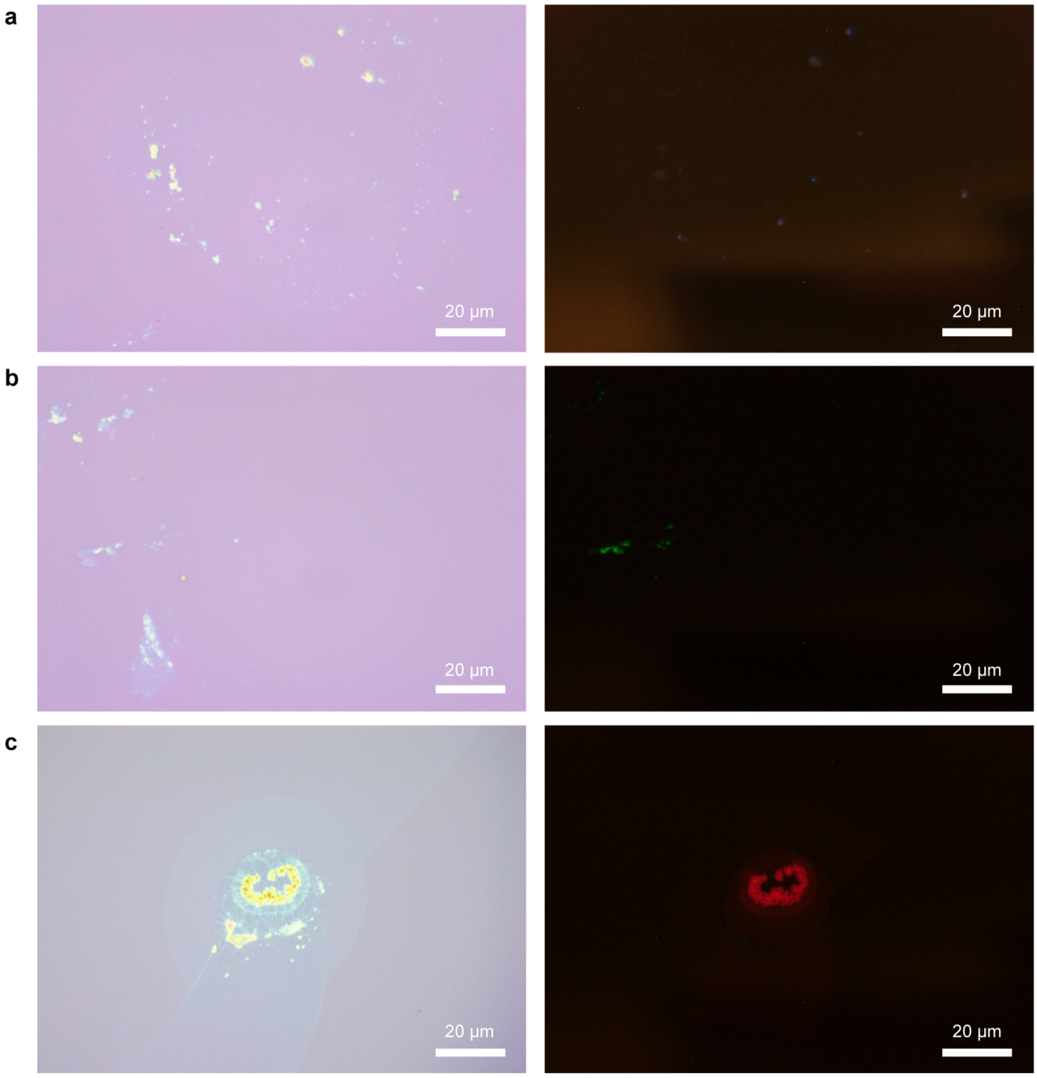

Figure S17. OM (left) and PL-OM (right) images of the a) $(PEA)_2PbBr_4$, b) $(PEA)_2PbI_4$, c) $(PEA)_2SnI_4$, materials prepared on a Si/SiO_2 substrate based on a spin-coating method.

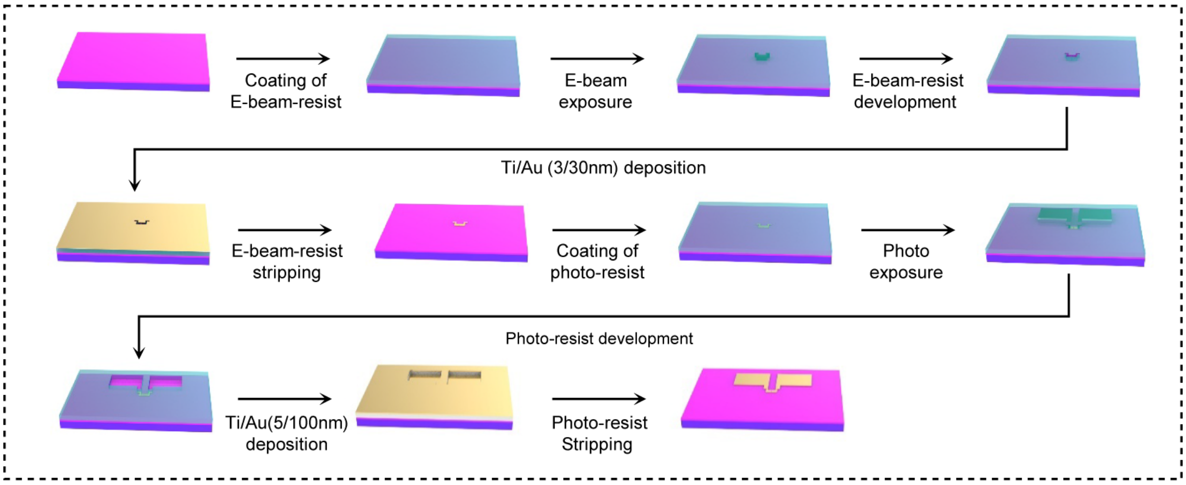

Figure S18. Schematic images of the electrodes and contact pads fabrication via e-beam and photo-lithography process.

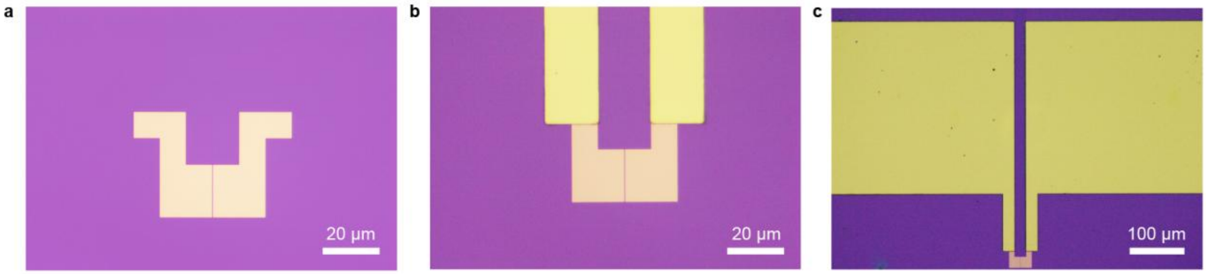

Figure S19. a) OM image of electrodes. b) OM image of the connection part between the electrode and the contact pad. c) OM images of overall electrode and contact pads.

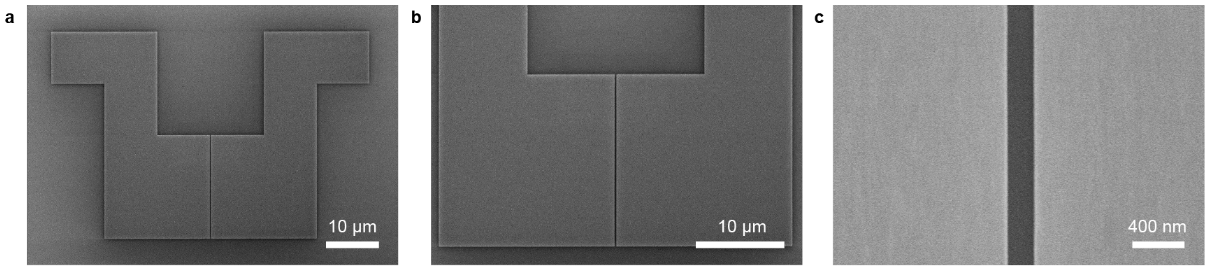

Figure S20. a) SEM image of electrodes and b) enlarged image. c) SEM image of electrode gap.

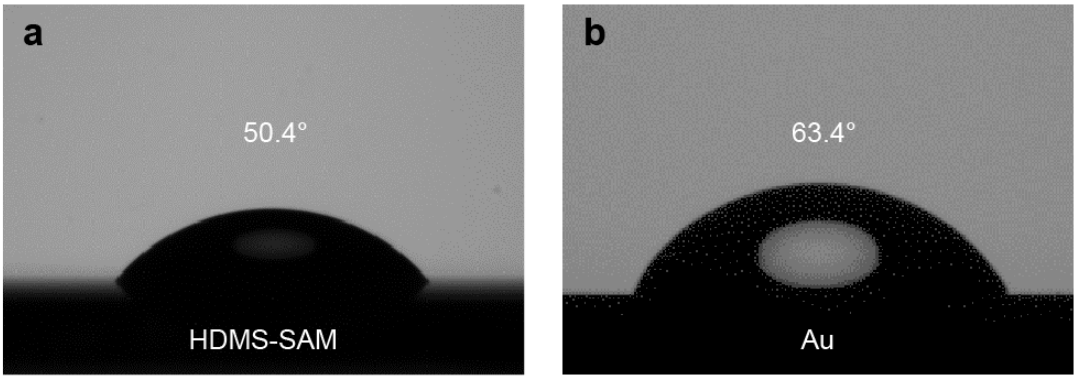

Figure S21. Contact angle measurements on a) HDMS-SAM substrate and b) Au electrode.

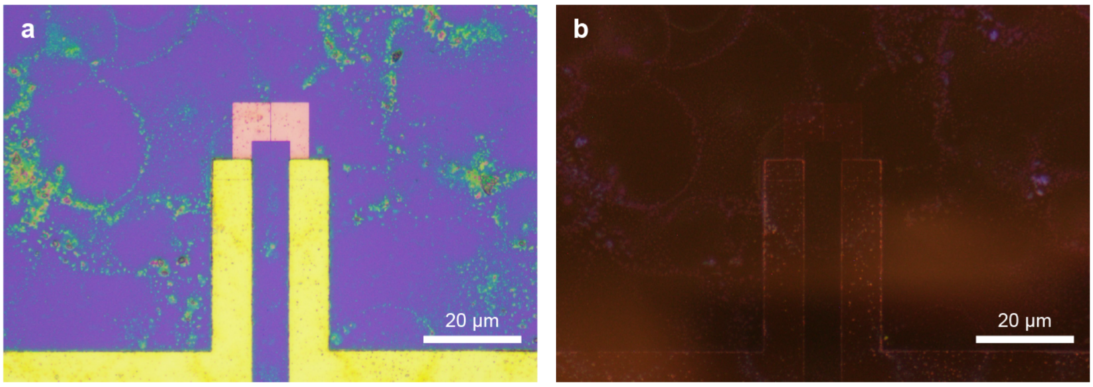

Figure S22. a) OM and b) OM-PL images of electrodes with $(PEA)_2PbBr_4$ nanocrystals on electrodes.

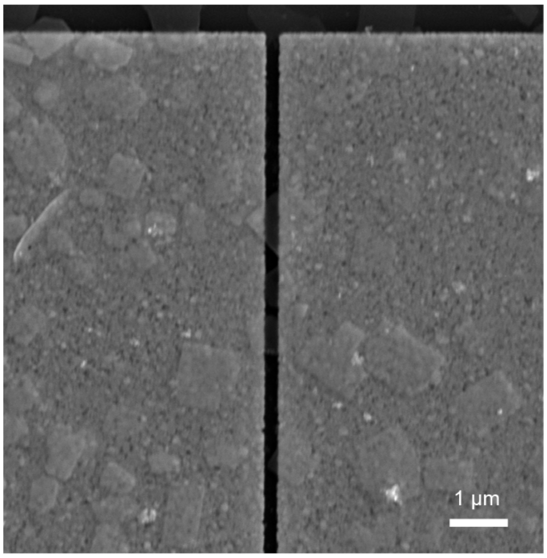

Figure S23. Gray-colored SEM images of $(PEA)_2PbBr_4$ single nanocrystals on electrodes.

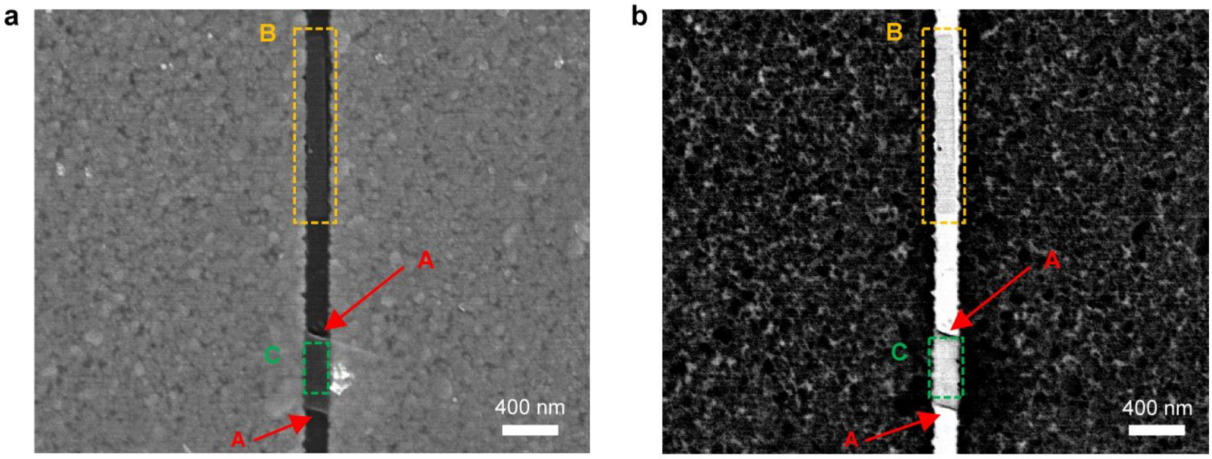

Figure S24. a) SEM image and b) contrast-adjusted SEM image of $(PEA)_2PbBr_4$ single nanocrystals on electrodes.

Supporting Note 1

Figure S24a shows an SEM image of two $(PEA)_2PbBr_4$ crystals located between the electrodes, synthesized using the USSC process. In this image, the region labeled "A" with a red arrow highlights a portion of the crystal at the electrode gap, where the crystal side on the electrodes exhibits downward bending. This feature is observed more clearly in Figure S24b, where the contrast of the SEM image has been adjusted. However, the majority of crystals did not show such bending, probably due to the small nanoscale gap minimizing crystal deformation.

Additionally, Figure. S24a displays a crystal synthesized within the electrode gap, highlighted by the orange dashed rectangle labeled "B." The crystal's image appears less dark compared to the inner region of the crystal at the electrode gap, marked with the green dashed rectangle labeled "C" in Figure. S24b. This contrast difference indicates a height variation of the crystal within the electrode gap, further supporting the hypothesis that the crystal is suspended between the electrodes.

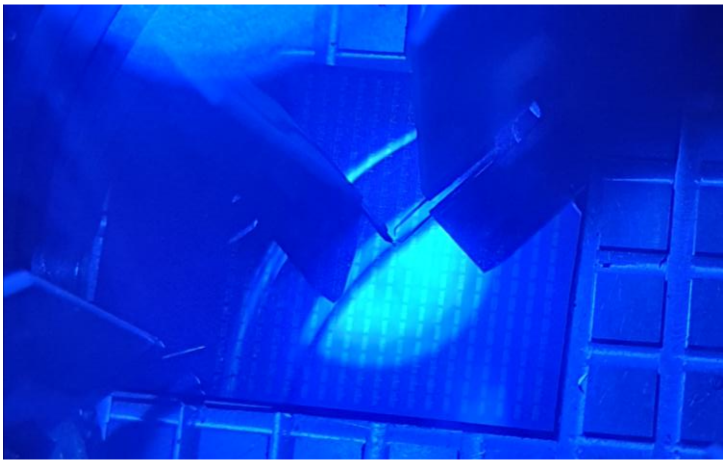

Figure S25. Photograph image of device measurement system.

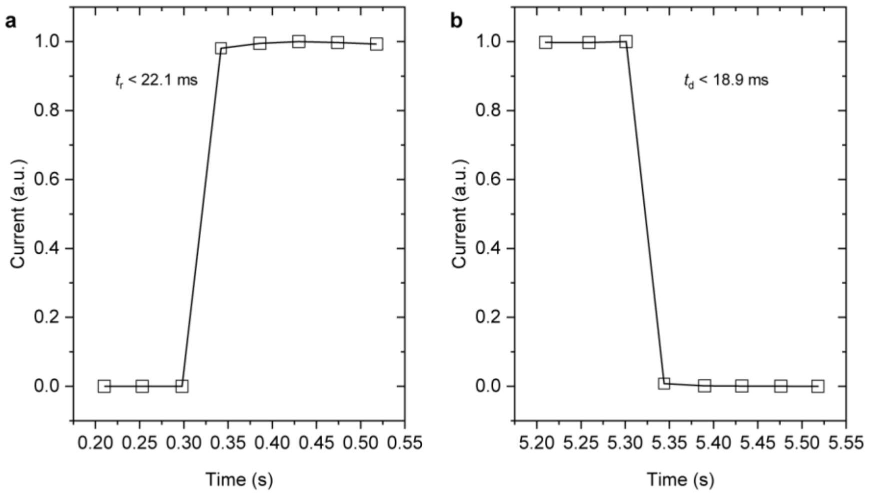

Figure S26. a) Rise and b) decay time estimation of 2D perovskite PDs under 365 nm-UV light illumination (Bias: 5 V, Light intensity: 37.5 μW/cm^2).

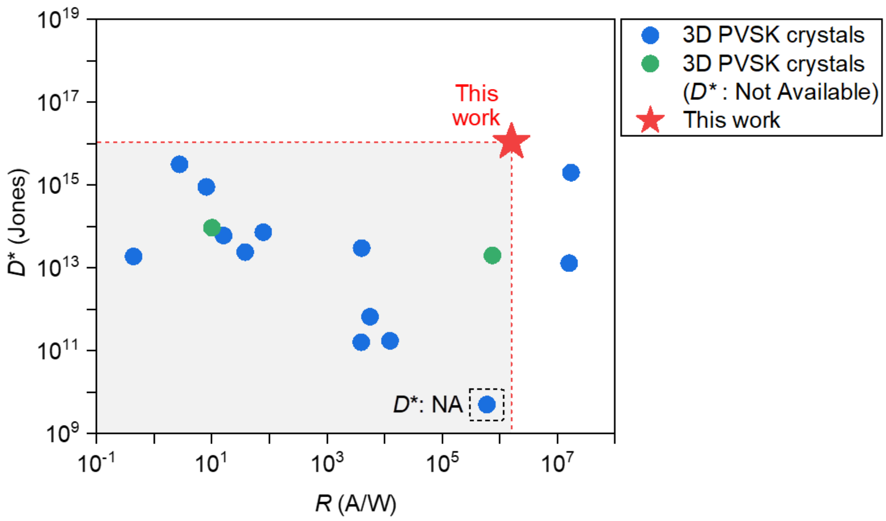

Figure S27. Comparison of the reported 3D perovskite materials-based PDs with those in our work with respect to the D^* and R. Related references are provided in the Supplementary references 31–44. Related references are provided in **Table S3**, Supporting Information.

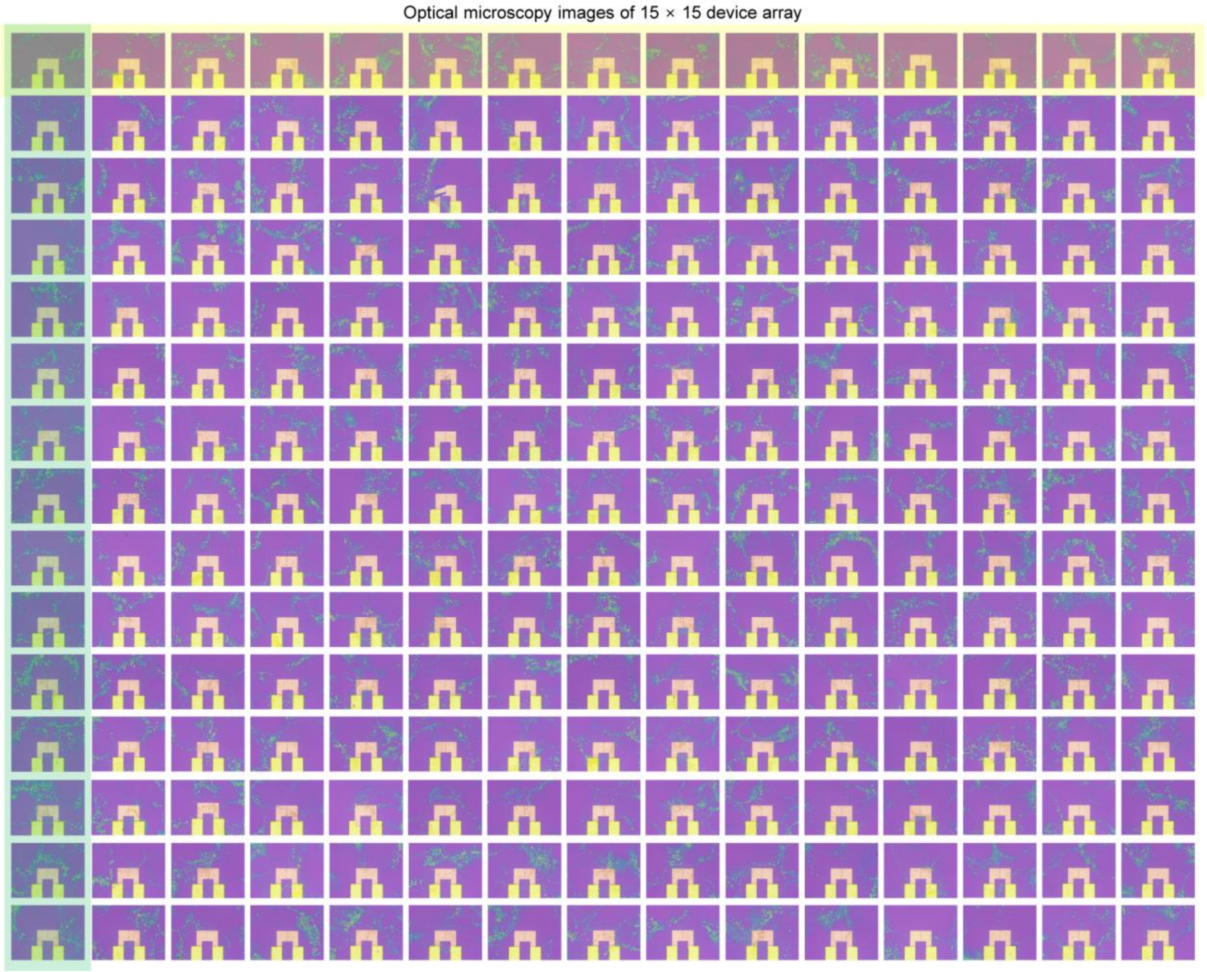

Figure S28. OM images of the 225 (up to 15×15 ea) PDs with a crystal placed between electrodes.

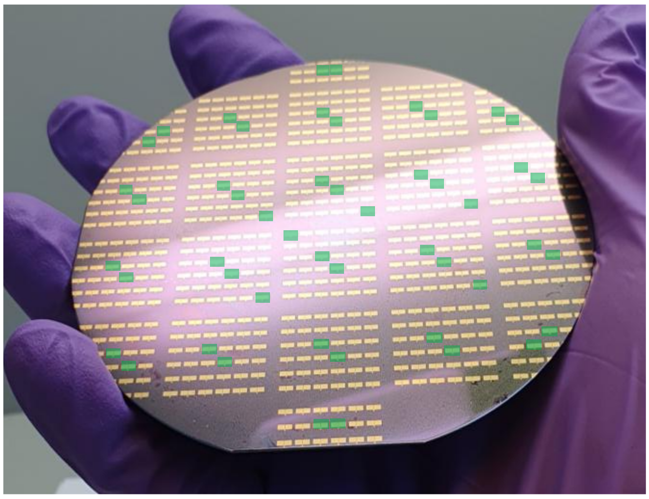

Figure S29. Schematic illustration of the fabricated 4-inch wafer scale PD arrays and selected 50 PDs (marked in green) for statistical analysis.

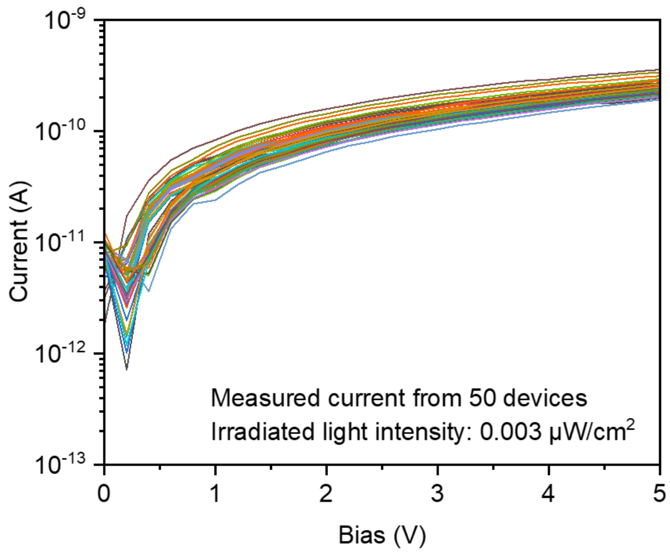

Figure S30. Individual *I-V* curves of the 50 PDs from PD array (λ = 365 nm, intensity 0.003 $\mu W/cm^2$).

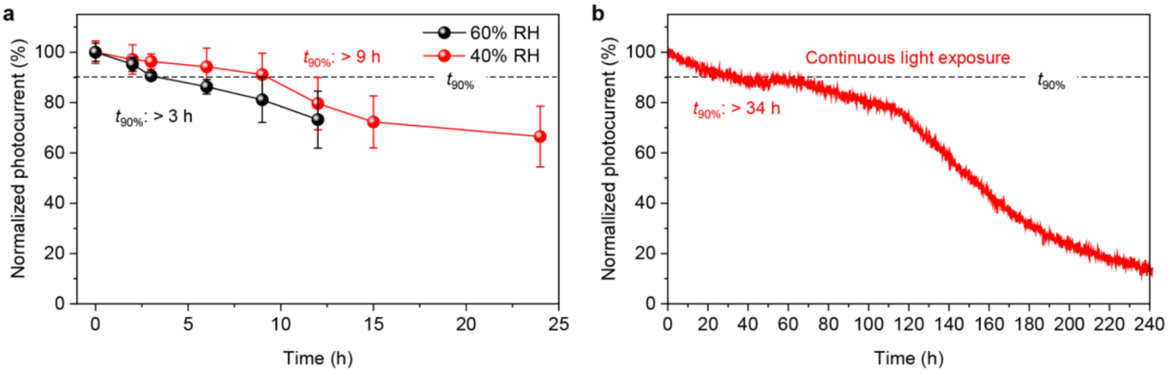

Figure S31. a) Humidity stability tests of the PDs in 40 and 60 RH% chambers at a room temperature. b) Photocurrent continuous tracking of the PDs under continuous UV light illumination in vacuum chamber at a room temperature. (λ = 365 nm, intensity 37.5 μW/cm^2)

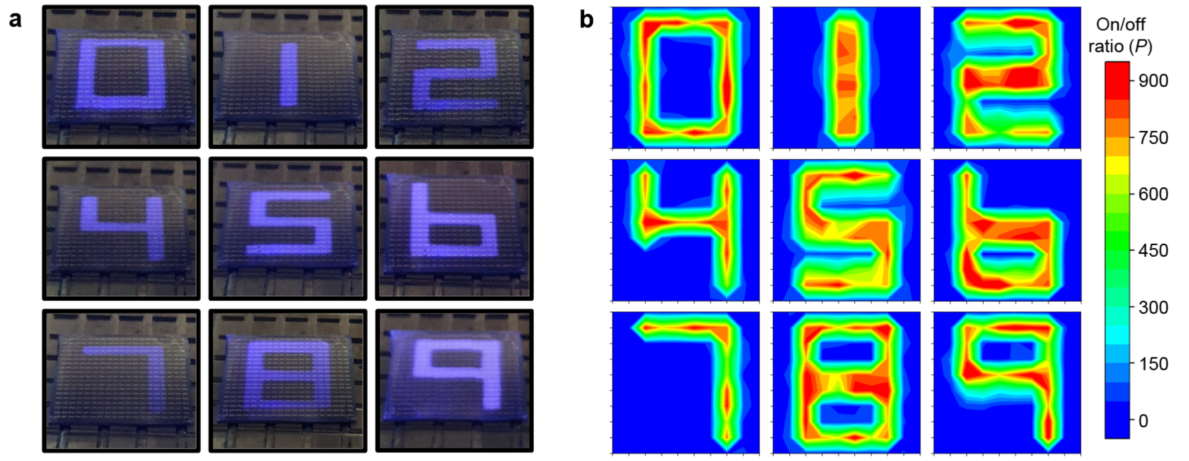

Figure S32. a) photograph images and b) corresponding spatial photo-sensing mapping results of 19×19 $(PEA)_2PbBr_4$ nanocrystal PD Array under number '0, 1, 2, 4, 5, 6, 7, 8, 9'-shaped light illumination.

Table S1. Averages (Ave.), standard deviations (Std.), and relative standard deviations of sizes of $(PEA)_2PbBr_4$ 2D perovskite crystals synthesized by USSC process based on 0.5 ml/min spray nozzle flow rates and conventional solvent drying methods.

Method	Ave. (nm)	Std. (nm)	Std./Ave. (%)
USSC	462	114	24.7
Conventional solvent drying	3886	2972	76.5

Table S2. Number of crystals per 1 cm^2.

	Dispensing rate (ml/min)				
	0.1	0.2	0.5	0.75	1.0
# of Crystals per 1 cm^2	1.00×10^{15}	4.84×10^{15}	7.19×10^{11}	2.25×10^{11}	4.77×10^{11}
	**Types of substrates**				
	SiO_2 Wafer	Glass	ITO	Al foil	Teflon
# of Crystals per 1 cm^2 (Dispensing rate : 0.5 ml/min)	7.19×10^{11}	7.69×10^{11}	7.74×10^{11}	5.70×10^{11}	1.77×10^{11}

Table S3. Comparison of the device performance parameters of 2D perovskite PDs reported in previous studies and this work.

**Material**	***R*** **(A/W)**	***D**** **(Jones)**	**λ (nm)**	**Intensity (μW/cm²)**	t_{Rise}/t_{Decay} **(μs)** (10%↔90%)	**Electrode gap (μm)**	**Bottom /Top**	**PVSK Form /Synthesis method**	**Ref.**
$Cs_2AgBiBr_6$	4.88	1.20E+13	550	0.07	8/8.8 (NA)	NA	Top	Crystal/ Solution Transfer	[1]
$(BA)_2PbI_4$	1.53	2.17E+11	546	1.9	131000/99000	NA	Vertical	Crystal/ Exfoliation	[2]
$(PEA)_2PbI_4$	0.46	1.20E+13	517	0.71	486/540	NA	Vertical	Crystal/ Exfoliation	[3]
$(PEA)_2PbI_4$	139.6	1.89E+15	462	0.033	21/37	20	Top	Crystal/ Solution Transfer	[4]
$(PEA)_2PbI_4$	98.17	1.62E+15	460	0.08	64/52	20	Top	Crystal/ Confinement	[5]
$(F\text{-}PEA)_2PbI_4$	2200	2.40E+18	514	0.000003	NA	NA	Bottom	Crystal/ Confinement	[6]
$(F\text{-}PEA)_2PbI_4$	1100	5.50E+17	514	0.000002	0.0015/NA (NA)	3	Bottom	Crystal/ Confinement	[7]
VPEA based perovskite	1.6	2.17E+13	490	1.3	306/318 (NA)	10	Top	Film/ Confinement	[8]
$(PEA)_2Pb(I_{0.9}Br_{0.1})_4$	4.8	6.50E+13	473	0.02	NA	25	Top	Crystal/ Confinement	[9]
$(PEA)_2SnI_4$	14.57	1.73E+12	470	21	50000/~1500000 (0→80%/100%→30%)	100	Bottom	Film/ Spin-coating	[10]
$(PEA)_2SnI_4$	3290	2.06E+11	470	195.8	370000/3050000 (NA)	6	Top	Crystal/ Solution Direct	[11]
$(BA)_2PbBr_4$	2100	NA	470	10	NA	0.1	Top	Crystal/ Solution Direct	[12]
$(BA)_2PbBr_4$	0.0004	9.01E+10	447	0.5	NA	NA	Top	Crystal/ Solution Transfer	[13]
$(BA)_2PbBr_4$	0.0453	1.08E+12	400	2750	9700/8800 (NA)	~64	Top	Crystal/ Solution Transfer	[14]
$BDAPbI_4$	0.927	1.23E+11	462	0.009	150/570	22	Top	Crystal/ Solution Transfer	[15]

$(BA)_2FAPb_2I_7$	2.3	3.20E+12	405	0.25	9.74/8.91	20	Bottom	Film/ Spin-coating	[16]
$(PA)_2PbBr_4$	0.0912	6.08E+12	405	0.02	280/290	200	Top	Crystal/ Solution Transfer	[17]
$(PA)_2PbBr_4$	2.22	2.30E+13	405	0.005	1590/1660	NA	Top	Crystal/ Confinement	[18]
$(BA)_2CsPb_2Br_7$	0.0395	1.20E+12	405	0.04	302/368	10	Top	Crystal/ Solution Transfer	[19]
$(BA)_2FA_{0.4}MA_{0.6}Pb_2I_7$	0.01	1.00E+11	405	40	NA	NA	Top	Crystal/ Exfoliation	[20]
$(NEA)_2(MA)_4Pb_5I_{16}$	1520	NA	405	7.8	NA	30	Top	Film/ Spin-coating	[21]
$(EA)_4Pb_3Br_{10}$	0.437	2.55E+12	400	0.1	7200/8500	5	Top	Crystal/ Confinement	[22]
$(BDA_{0.7}(BA_2)_{0.3})(EA)_2Pb_3Br_{10}$	93	2.50E+15	360	0.12	370/920 (NA)	NA	Vertical	Film/ Spin-coating	[23]
$(MPA)_2PbCl_4$	0.002	1.20E+12	266	15000	150/150	NA	Vertical	Crystal/ Solution Transfer	[24]
$(BA)_4MA_3Pb_4I_{13}$	10	1.60E+13	White	4	383000/177000	50	Top	Crystal/ Solution Transfer	[25]
$(PEA)_2MA_2Pb_3I_{10}$	0.279	NA	White	60	NA	100	Top	Crystal/ Solution Transfer	[26]
$(PEA)_2PbBr_4$	0.025	2.54E+11	395	13500	15.66/15.45	NA	Vertical	Film/ Spin-coating	[27]
$(PEA)_2PbBr_4$	0.0083	2.50E+12	380	0.045	145000/672000	20	Bottom	Crystal/ Solution Direct	[28]
$(PEA)_2PbBr_4$	31.1	4.03E+13	365	0.02	NA	5	Top	Crystal/ Solution Direct	[29]
$(PEA)_2PbBr_4$	0.0315	1.55E+13	365	3	410/370	40	Top	Crystal/ Solution Transfer	[30]
$(PEA)_2PbBr_4$	1600000	1.10E+16	365	0.003	22100/18900	0.2	Bottom	Crystal/ Spray Coating	**This work**

NA: Not available, F-PEA: Fluorophenylethylammonium, VPEA: Vinylphenylethylammonium, EA: Ethylammonium, BDA: Butanediammonium, MA: Methylammonium, FA: Formamidinium, MPA: Methylphenethylammonium, NEA: (S, R)-1-(2-Naphthyl)-ethylammonium

Table S4. Comparison of the device performance parameters of 3D perovskite PDs reported in previous studies and this work.

Material	***R*** (A/W)	***D**** (Jones)	**λ** (nm)	**Intensity** (μW/cm^2)	t_{Rise}/t_{Decay} (μs) (10%↔90%)	**Electrode gap (μm)**	**Bottom /Top**	**PVSK form / Synthesis method**	**Ref.**
$MAPbI_3$	2.75	3.17E+15	670	10	19000/19000	NA	Bottom	Wire/ Solution Transfer	[31]
$MAPbI_3$	12500	1.73E+11	532	0.100	0.34/0.42	25	Top	Crystal/ Solution Direct	[32]
$MAPbI_3$	78.70	7.23E+13	460	10	14/14	2.5	Bottom	Crystal/ Solution Direct	[33]
$MAPbI_3$	17300000	2.00E+15	White	0.133	NA/879000 (100%→30%)	3	Bottom	Film	[34]
$MAPbI_3$	38	2.40E+13	White	4	773000/385000	50	Top	Crystal/ Solution Transfer	[25]
$MAPbBr_3$	16	6.00E+13	525	12	43/36	40	Top	Crystal/ Solution Transfer	[35]
$MAPbBr_3$	5600	6.59E+11	514	0.08	3.2/9.2 (N/A)	NA	Top	Crystal/ Confinement	[36]
$MAPbBr_3$	4000	3.00E+13	450	0.000002 μW	~25/~25	5	Bottom	Crystal Film/ Solution Direct	[37]
$MAPbBr_3$	16000000	1.30E+13	405	0.000000025 μW	88/930	NA	Vertical	Crystal/ Confinement	[38]
$CsPbBr_3$	10.10	9.35E+13	405	13400	0.000023/NA (10%→90%)	NA	Top	Film/ Spin-coating	[39]
$CsPbBr_3$	0.44	1.88E+13	405	0.009	28/270 (NA)	NA	Vertical	Film/ Spin-coating	[40]
$MAPbBr_2I$	600000	NA	405	0.001	~120000/~750000 (0→70%/100%→30%)	10	Top	Crystal/ Solution Direct	[41]
$CsPbCl_3$	748000	2.00E+13	390	6.72	300000/3500000 (NA)	4	Top	Crystal/ Solution Direct	[42]
$CsPbCl_3$	8.10	9.00E+14	365	100	28000/31000 (NA)	NA	Top	Film/ Spin-coating	[43]
$CsPbBr_{1.5}I_{1.5}$	3946	1.60E+11	White	45000	116000/147000	NA	Top	Crystal Film/ Spin-coating	[44]
$(PEA)_2PbBr_4$	1600000	1.10E+16	365	0.003	22100/18900	0.2	Bottom	Crystal/ Spray Coating	**This work**

Supplementary Video

Video S1. Optical microscope video for liquid bridge flow of $(PEA)_2PbBr_4$ 2D PVSK precursor droplet via spray coating process.

Video S2. Large-scale spray coating synthesis of $(PEA)_2PbBr_4$ 2D PVSK nanocrystals for photodetector array.

Supporting References

[1] D. Hao, D. Liu, S. Zhang, L. Li, B. Yang, J. Huang, *Adv. Opt. Mater.* **2022**, *10*, 2100786.
[2] H. Wang, L. Li, J. Ma, J. Li, D. Li, *J. Mater. Chem. C* **2021**, *9*, 11085.
[3] Y. Tu, Y. Xu, J. Li, Q. Hao, X. Liu, D. Qi, C. Bao, T. He, F. Gao, W. Zhang, *Small* **2020**, *16*, 2005626.
[4] Y. Liu, H. Ye, Y. Zhang, K. Zhao, Z. Yang, Y. Yuan, H. Wu, G. Zhao, Z. Yang, J. Tang, Z. Xu, S. (Frank) Liu, *Matter* **2019**, *1*, 465.
[5] Y. Liu, Y. Zhang, Z. Yang, H. Ye, J. Feng, Z. Xu, X. Zhang, R. Munir, J. Liu, P. Zuo, Q. Li, M. Hu, L. Meng, K. Wang, D.-M. Smilgies, G. Zhao, H. Xu, Z. Yang, A. Amassian, J. Li, K. Zhao, S. Liu, *Nat. Commun.* **2018**, *9*, 5302.
[6] K. J. Riisnaes, M. Alshehri, I. Leontis, R. Mastria, H. T. Lam, L. De Marco, A. Coriolano, M. F. Craciun, S. Russo, *ACS Appl. Mater. Interfaces* **2024**, *16*, 31399.
[7] R. Mastria, K. J. Riisnaes, A. Bacon, I. Leontis, H. T. Lam, M. A. S. Alshehri, D. Colridge, T. H. E. Chan, A. De Sanctis, L. De Marco, L. Polimeno, A. Coriolano, A. Moliterni, V. Olieric, C. Giannini, S. Hepplestone, M. F. Craciun, S. Russo, *Adv. Funct. Mater.* **2024**, *34*, 2401903.
[8] Y. Zhao, X. Yin, Z. Gu, M. Yuan, J. Ma, T. Li, L. Jiang, Y. Wu, Y. Song, *Adv. Funct. Mater.* **2023**, *33*, 2306199.
[9] X. Zhang, D. Zhao, Z. Huo, J. Sun, Y. Hu, Z. Lou, Y. Hou, F. Teng, Q. Cui, *Opt. Mater.* **2021**, *117*, 111074.
[10] H. Wang, Y. Chen, E. Lim, X. Wang, S. Yuan, X. Zhang, H. Lu, J. Wang, G. Wu, T. Lin, S. Sun, J. Wang, Y. Zhan, H. Shen, X. Meng, J. Chu, *J. Mater. Chem. C* **2018**, *6*, 12714.
[11] L. Qian, Y. Sun, M. Sun, Z. Fang, L. Li, D. Xie, C. Li, L. Ding, *J. Mater. Chem. C* **2019**, *7*, 5353.
[12] Z. Tan, Y. Wu, H. Hong, J. Yin, J. Zhang, L. Lin, M. Wang, X. Sun, L. Sun, Y. Huang, K. Liu, Z. Liu, H. Peng, *J. Am. Chem. Soc.* **2016**, *138*, 16612.
[13] E. Choi, Y. Zhang, A. M. Soufiani, M. Lee, R. F. Webster, M. E. Pollard, P. J. Reece, W. Lee, J. Seidel, J. Lim, J.-H. Yun, J. S. Yun, *Npj 2D Mater. Appl.* **2022**, *6*, 1.
[14] S. Wang, Y. Chen, J. Yao, G. Zhao, L. Li, G. Zou, *J. Mater. Chem. C* **2021**, *9*, 6498.
[15] Y. Zhang, Y. Liu, Z. Xu, Z. Yang, S. (Frank) Liu, *Small* **2020**, *16*, 2003145.
[16] T. Wang, D. Zheng, J. Zhang, J. Qiao, C. Min, X. Yuan, M. Somekh, F. Feng, *Adv. Funct. Mater.* **2022**, *32*, 2208694.
[17] C. Zhang, H. Xiao, Q. Guan, T. Zhu, L. Liang, R. Li, H. Ye, X. Niu, J. Luo, *J. Mater. Chem. C* **2023**, *11*, 5116.
[18] K. Dong, H. Zhou, Z. Gao, M. Xu, L. Zhang, S. Zhou, H. Cui, S. Wang, C. Tao, W. Ke, F. Yao, G. Fang, *Adv. Funct. Mater.* **2024**, *34*, 2306941.
[19] J. Wang, Y. Liu, S. Han, Y. Ma, Y. Li, Z. Xu, J. Luo, M. Hong, Z. Sun, *Sci. Bull.* **2021**, *66*, 158.
[20] G. Liu, J. Liu, Z. Fan, X. He, K. Luo, Q. Ye, C. Liao, *Phys. Status Solidi RRL – Rapid Res. Lett.* **2021**, *15*, 2100099.
[21] T. Liu, W. Shi, W. Tang, Z. Liu, B. C. Schroeder, O. Fenwick, M. J. Fuchter, *ACS Nano* **2022**, *16*, 2682.
[22] R. Ding, Y. Lyu, Z. Wu, F. Guo, W. F. Io, S.-Y. Pang, Y. Zhao, J. Mao, M.-C. Wong, J. Hao, *Adv. Mater.* **2021**, *33*, 2101263.
[23] L. Guo, Y. Qi, Z. Wu, X. Yang, G. Yan, R. Cong, L. Zhao, W. Zhang, S. Wang, C. Pan, Z. Yang, *Adv. Mater.* **2023**, *35*, 2301705.
[24] X. Zhang, W. Weng, L. Li, H. Wu, Y. Yao, Z. Wang, X. Liu, W. Lin, J. Luo, *Small* **2021**, *17*, 2102884.
[25] K. Wang, C. Wu, D. Yang, Y. Jiang, S. Priya, *ACS Nano* **2018**, *12*, 4919.

[26] Y. Zhang, M. Sun, N. Zhou, B. Huang, H. Zhou, *J. Phys. Chem. Lett.* **2020**, *11*, 7610.
[27] Y. Subramaniam, K. L. Woon, *Synth. Met.* **2023**, *293*, 117261.
[28] F. Lédée, A. Ciavatti, M. Verdi, L. Basiricò, B. Fraboni, *Adv. Opt. Mater.* **2022**, *10*, 2101145.
[29] Y. H. Lee, J. Y. Park, P. Niu, H. Yang, D. Sun, L. Huang, J. Mei, L. Dou, *ACS Nano* **2023**, *17*, 13840.
[30] Y. Zhang, Y. Liu, Z. Xu, H. Ye, Q. Li, M. Hu, Z. Yang, S. (Frank) Liu, *J. Mater. Chem. C* **2019**, *7*, 1584.
[31] Y. H. Lee, I. Song, S. H. Kim, J. H. Park, S. O. Park, J. H. Lee, Y. Won, K. Cho, S. K. Kwak, J. H. Oh, *Adv. Mater.* **2020**, *32*, 2002357.
[32] W. Deng, L. Huang, X. Xu, X. Zhang, X. Jin, S.-T. Lee, J. Jie, *Nano Lett.* **2017**, *17*, 2482.
[33] V. Venugopalan, R. Sorrentino, P. Topolovsek, D. Nava, S. Neutzner, G. Ferrari, A. Petrozza, M. Caironi, *Chem* **2019**, *5*, 868.
[34] P.-H. Chang, S.-Y. Liu, Y.-B. Lan, Y.-C. Tsai, X.-Q. You, C.-S. Li, K.-Y. Huang, A.-S. Chou, T.-C. Cheng, J.-K. Wang, C.-I. Wu, *Sci. Rep.* **2017**, *7*, 46281.
[35] Y. Liu, Y. Zhang, K. Zhao, Z. Yang, J. Feng, X. Zhang, K. Wang, L. Meng, H. Ye, M. Liu, S. (Frank) Liu, *Adv. Mater.* **2018**, *30*, 1707314.
[36] H. Jing, R. Peng, R.-M. Ma, J. He, Y. Zhou, Z. Yang, C.-Y. Li, Y. Liu, X. Guo, Y. Zhu, D. Wang, J. Su, C. Sun, W. Bao, M. Wang, *Nano Lett.* **2020**, *20*, 7144.
[37] M. I. Saidaminov, V. Adinolfi, R. Comin, A. L. Abdelhady, W. Peng, I. Dursun, M. Yuan, S. Hoogland, E. H. Sargent, O. M. Bakr, *Nat. Commun.* **2015**, *6*, 8724.
[38] Z. Yang, Y. Deng, X. Zhang, S. Wang, H. Chen, S. Yang, J. Khurgin, N. X. Fang, X. Zhang, R. Ma, *Adv. Mater.* **2018**, *30*, 1704333.
[39] K. Shen, H. Xu, X. Li, J. Guo, S. Sathasivam, M. Wang, A. Ren, K. L. Choy, I. P. Parkin, Z. Guo, J. Wu, *Adv. Mater.* **2020**, *32*, 2000004.
[40] G. Cen, Y. Liu, C. Zhao, G. Wang, Y. Fu, G. Yan, Y. Yuan, C. Su, Z. Zhao, W. Mai, *Small* **2019**, *15*, 1902135.
[41] Y. Wang, Y. Zhang, Y. Lu, W. Xu, H. Mu, C. Chen, H. Qiao, J. Song, S. Li, B. Sun, Y.-B. Cheng, Q. Bao, *Adv. Opt. Mater.* **2015**, *3*, 1389.
[42] M. Gong, R. Sakidja, R. Goul, D. Ewing, M. Casper, A. Stramel, A. Elliot, J. Z. Wu, *ACS Nano* **2019**, *13*, 1772.
[43] D. Li, D. Zhou, W. Xu, X. Chen, G. Pan, X. Zhou, N. Ding, H. Song, *Adv. Funct. Mater.* **2018**, *28*, 1804429.
[44] A. Mandal, A. Ghosh, D. Ghosh, S. Bhattacharyya, *ACS Appl. Mater. Interfaces* **2021**, *13*, 43104.
